# Molecular Mechanism Biomarkers Predict Diagnosis in Schizophrenia and Schizoaffective Psychosis, with Implications for Treatment

**DOI:** 10.3390/ijms242115845

**Published:** 2023-10-31

**Authors:** Stephanie Fryar-Williams, Graeme Tucker, Jörg Strobel, Yichao Huang, Peter Clements

**Affiliations:** 1 Youth in Mind Research Institute, Unley, SA 5061, Australia; 2 The Queen Elizabeth Hospital, Woodville, SA 5011, Australia; 3 Basil Hetzel Institute for Translational Health Research, Woodville, SA 5011, Australia; 4 Department of Nanoscale BioPhotonics, Faculty of Health and Medical Sciences, School of Biomedicine, The University of Adelaide, Adelaide, SA 5000, Australia; 5 Department of Public Health, Faculty of Health and Medical Sciences, Adelaide Medical School, The University of Adelaide, Adelaide, SA 5000, Australia; graeme.tucker@adelaide.edu.au; 6 Department of Psychiatry, Faculty of Health and Medical Sciences, Adelaide Medical School, The University of Adelaide, Adelaide, SA 5000, Australia; jorg.strobel@adelaide.edu.au; 7 Waite Research Institute, The University of Adelaide, Urrbrae, SA 5064, Australia; 8 Department of Paediatrics, Faculty of Health and Medical Sciences, Adelaide Medical School, The University of Adelaide, Adelaide, SA 5000, Australia

**Keywords:** biomarkers, psychosis, schizophrenia, bipolar, vitamin B6, riboflavin, *MTHFR* C677T genotype, methylation, phenotypes, treatment applications

## Abstract

Diagnostic uncertainty and relapse rates in schizophrenia and schizoaffective disorder are relatively high, indicating the potential involvement of other pathological mechanisms that could serve as diagnostic indicators to be targeted for adjunctive treatment. This study aimed to seek objective evidence of methylenetetrahydrofolate reductase *MTHFR* C677T genotype-related bio markers in blood and urine. Vitamin and mineral cofactors related to methylation and indolamine-catecholamine metabolism were investigated. Biomarker status for 67 symptomatically well-defined cases and 67 asymptomatic control participants was determined using receiver operating characteristics, Spearman’s correlation, and logistic regression. The 5.2%-prevalent *MTHFR* 677 TT genotype demonstrated a 100% sensitive and specific case-predictive biomarkers of increased riboflavin (vitamin B2) excretion. This was accompanied by low plasma zinc and indicators of a shift from low methylation to high methylation state. The 48.5% prevalent *MTHFR* 677 CC genotype model demonstrated a low-methylation phenotype with 93% sensitivity and 92% specificity and a negative predictive value of 100%. This model related to lower vitamin cofactors, high histamine, and HPLC urine indicators of lower vitamin B2 and restricted indole-catecholamine metabolism. The 46.3%-prevalent CT genotype achieved high predictive strength for a mixed methylation phenotype. Determination of *MTHFR* C677T genotype dependent functional biomarker phenotypes can advance diagnostic certainty and inform therapeutic intervention.

## 1. Introduction

It is recognized that schizophrenia arises from a complex range of different pathological factors, which include developmental risk factors, environmental effects, and genetic traits [1]. These factors accumulate and interact to produce a variety of symptoms in adolescence and youth [2]. Features of schizophrenia include delusions, loss of judgement and insight, cognitive impairment, altered volition, heightened, or dulled emotional reactivity and disorganized behavior. If such symptoms are accompanied by significant changes in mood, the condition is often termed “schizoaffective” disorder [3]. Initial symptoms may include anxiety or depressed mood or hallucinations, which are insufficient to be allocated a diagnosis with any certainty. The course and outcome of symptom development may also vary considerably, with symptoms resolving or exacerbating over time or merging with other mental illness states. Abnormal mental states may resolve in some cases or episodically flare up or progress in a chronic debilitating manner, leading to misdiagnosis or diagnostic dilemma because the symptoms vary over time [4]. These difficulties are often associated with poor response to medication and frequent disrupting relapses [5]. In this context, it is recognized that diagnostic uncertainty and relapse rates in schizophrenia and schizoaffective disorder are relatively high [6], indicating the potential involvement of other pathological mechanisms that could serve as diagnostic indicators and be targeted for treatment. Moreover, the stressful effect of diagnostic uncertainty and frequent relapse is often fed back to the patient and the health care system, expressing itself as anxiety, impoverished social relations, and impaired individual and family productivity [4,7]. Against this background of difficulty, there are no objective biochemical or other phenotypes for subtyping and treating psychotic psychiatric conditions such as schizophrenia and schizoaffective disorder. Thus, there is a need for another assessment paradigm to be deployed—one which can objectively identify biochemical measures with sufficient sensitivity and specificity to detect and screen for a diagnosis of psychosis, inform about its phenotypic subtype, and promote understanding of psychosis at a fundamental biological level. Such biomarkers can assist clinicians to monitor illness progression, prevent relapse, overcome treatment resistance, and inform individual clinical management decisions [8]. Ideally, such a paradigm can stand alone or provide objective support for traditional symptom-based diagnostic assessment methods.

In search of a paradigm, this study sought to find a novel set of biochemical markers with sufficient power for the diagnosis and prediction of outcomes in schizophrenia and schizoaffective psychosis. The methylenetetrahydrofolate reductase gene (*MTHFR* C677T gene) codes for the methylenetetrahydrofolate reductase (MTHFR) enzyme, which converts 5, 10-methylenetetrahydrofolate to 5-methyltetrahydrofolate (5-MTHF). 5-MTHF is an important methyl donor molecule and the MTHFR enzyme is the rate-limiting enzyme for the biochemical process of methylation [9]. The homozygous polymorphism form of this *MTHFR* C677T gene is the *MTHFR* 677 TT genotype, which has been implicated in multiple pathological conditions, including pre-eclampsia, folate deficient fetal neural tube defects, colon cancer, and depression and schizophrenia in adults [10]. The MTHFR enzyme provides methyl groups for the methylation cycle, and this cycle provides methyl groups for DNA and histone methylation for the suppression of gene transcription [11]. It was therefore reasoned that stratifying schizophrenia and schizoaffective psychosis data across *MTHFR* C677T genotypes might uncover deeper methylation-related meanings. It was also hypothesized that this approach might make better sense of biochemical data and allow the resolution of inconsistent findings previously reported in schizophrenia research related to this gene [12,13]. The findings of this study validate this hypothesis by demonstrating that *MTHFR* C677T genotypes do indeed differentiate data into functionally meaningful methylation phenotypes. Single or grouped genotype-related biomarkers within *MTHFR* C677T genotype dependent regression models were discovered to strongly predict a diagnosis of schizophrenia and schizoaffective disorder. These biomarkers consist of riboflavin (vitamin B2) excretion markers and other blood-borne vitamin B6, vitamin B12, and vitamin D biomarkers. Together with copper and zinc trace minerals, these act as cofactors in molecular pathways relating to the MTHFR enzyme. Since the activity of this enzyme is determined by its *MTHFR* C677T genotype, the level of this enzyme, combined with cofactor availability, alters the dynamics of indole-catecholamines, glutathione, and intermediate substances in a wide range of molecular pathways. The formation of riboflavin’s flavo-mononucleotide (FMN) and flava-dinucleotide (FAD) products are prerequisite cofactors for MTHFR enzyme activity and for S-adenosyl methionine (SAMe) synthesis. SAMe is a critical methyl donor for indole-catecholamine metabolism and for DNA methylation. Since DNA methylation modulates the expression of other genes relevant to schizophrenia, the relationship of the MTHFR enzyme activity to SAMe availability has wide implications for the function of multiple genes and the field of epigenetics [14]. Although these biomarkers have potential to guide therapeutic interventions in schizophrenia and schizoaffective psychosis, there have been no studies performed that relate these specific biomarkers to treatment efficacy in these conditions. Neither is it clear how antipsychotic therapy affects the biomarkers concerned. We hope to investigate these issues alongside a treatment and validation study with wider biomarker selection in larger cohorts. Once validated, these biomarkers have potential to assist clinicians to monitor illness progression and remission, prevent relapses, and overcome treatment resistance. 

**Scheme 1:**
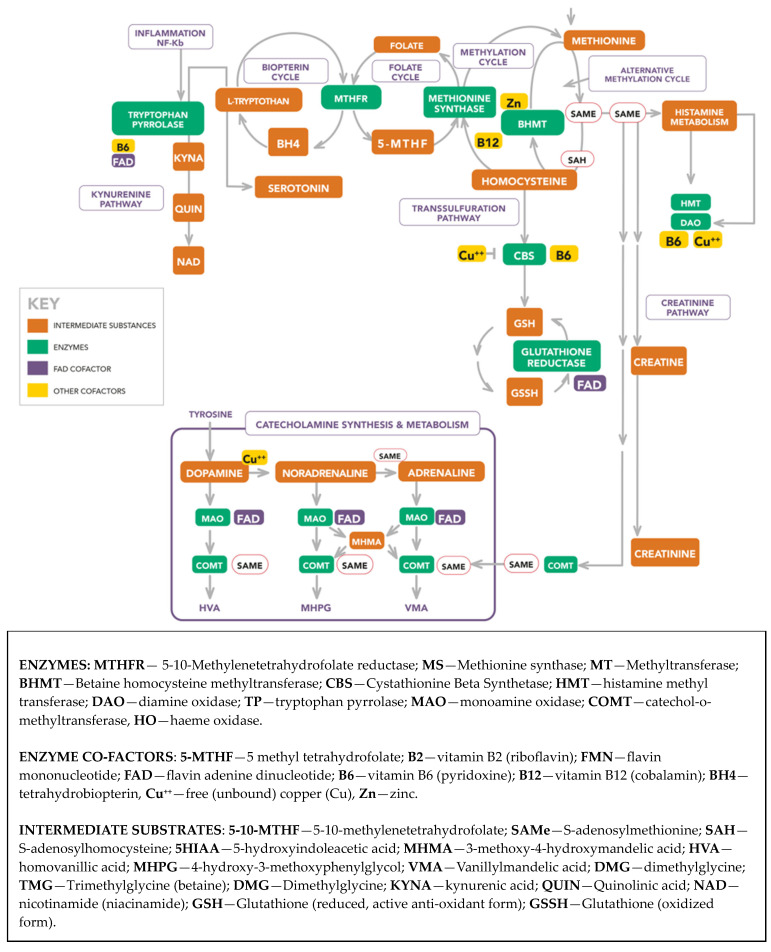
Extended biochemistry diagram.

Scheme 1 portrays the molecular landscape from which the biomarkers were selected and the dynamic interrelationships between the enzymes, cofactors, and intermediate substances in that landscape. A description of abbreviations used in this landscape is provided below Scheme 1. An extended Abbreviations section for molecules and methods used in this study is provided at end of this article and a more detailed description of the molecular landscape is available in a recent publication [15].

## 2. Results and Discussion

### 2.1. Summary of Data Characteristics

Participants diagnosed with first episode psychosis, established schizophrenia or schizoaffective disorder were recruited over three years of the study. Participants were drawn from a sample of 2487 persons associated with a general population of 22,000 persons in the northwest area of Adelaide, South Australia. After the confirmation of the inclusion criteria and pairing for gender and age, data analysis was conducted on 67 participants and 67 controls. Table 1 contains a sample of demographic data with further data supplied in the Supplementary Materials, Section S5.

### 2.2. Significance of the MTHFR 677 TT Genotype for Riboflavin Results 

Creatinine-related ROC characteristics for urine riboflavin (vitamin B2/creatinine ROC in Table 2) were 100% sensitive and specific for the homozygous *MTHFR* 677 TT polymorphism genotype, and this finding is supported by a strong correlative relationship between vitamin B2 excretion and case-certainty (*n*6, *rho* 0.829, *p* 0.042). The reason for this will be discussed in Section 2.2.1 below. In contrast, vitamin B2-related parameters for the heterozygous *MTHFR* 677 CT genotype and the *MTHFR* 677 CC-dependent genotype were neither sensitive nor specific for diagnosis.

**Figure 1:**
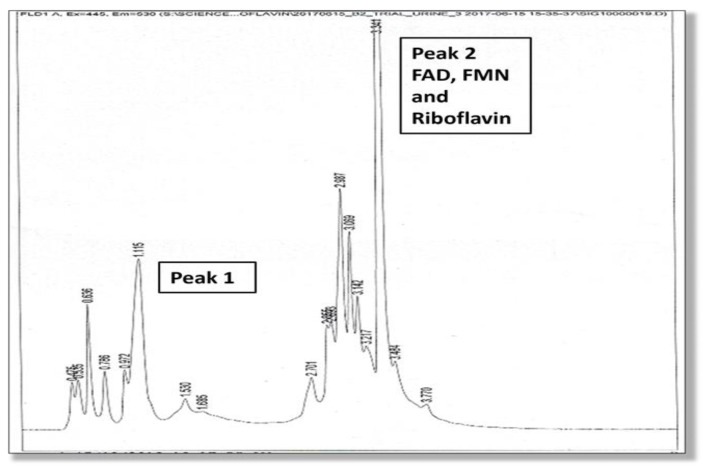
HPLC elution phenotype (associated with the *MTHFR* 677 TT genotype), where the height of Peak 2 (excretion of riboflavin, FMN and FAD) exceeds Peak 1 (excretion of presumed metabolites).

**Figure 2:**
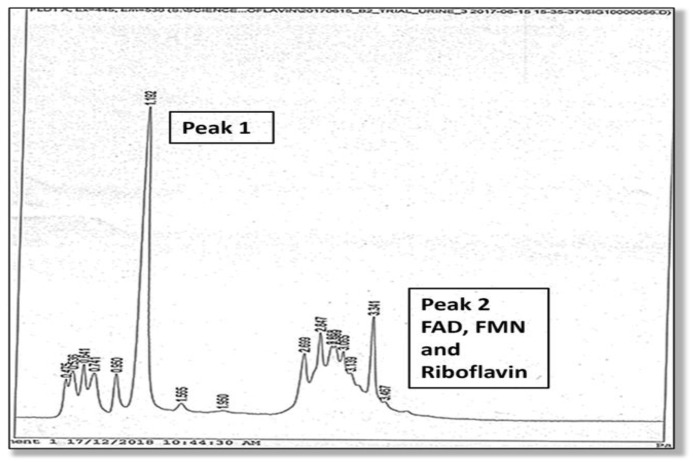
HPLC elution (associated with the *MTHFR* 677 CC genotype), where the height of Peak 1 (excretion of presumed metabolites) exceeds Peak 2 (excretion of riboflavin, FMN, and FAD).

A full description of the high-pressure liquid chromatography method used for the urine analysis of flavin molecules in this study is provided in the Supplementary Materials, Section S4. Two high performance liquid chromatography (HPLC) elution peaks were found and the amplitude and area under Peak 1 and Peak 2 were measured. The second peak (Peak 2) was identified as being riboflavin itself, and flavoprotein cofactors (FMN and FAD) were identified at the leading edge of this second riboflavin peak. Although Peak 1 was not directly identified due to lack of urine sample, it is expected to contain metabolites of riboflavin, such as hydroxy riboflavin. Another metabolite (lumiflavin) was not found in Peak 1 on follow up HPLC study. Many metabolites of riboflavin, such as 10-2′-hydroxyethyl flavin are competitive inhibitors of riboflavin cellular uptake. For instance, 10-2′-hydroxyethyl flavin inhibits the flavokinase phosphorylation of riboflavin to FMN [17]. These HPLC findings are shown in Figure 1 and Figure 2.

A linear relationship has been demonstrated in research between riboflavin levels and its urinary excretion levels and between riboflavin absorption and its tissue bioavailability [17,18,19,20]. Therefore, we understand that relatively higher levels of riboflavin excretion reflect adequate riboflavin absorption and bioavailability in plasma and tissue. However, since lower Peak 1 levels in urine reflect riboflavin degradation in tissue or by bacteria in the gastrointestinal tract [17,21], the lower urinary Peak 1 levels relative to Peak 2 riboflavin levels observed in this this TT genotype imply that riboflavin degradation is low and riboflavin is thereby conserved. Proposed mechanisms for this are discussed in Section 2.2.1 below. In this setting, vitamin B2 excretion ROC (at 95% confidence interval, adjusted for the Australian schizophrenia prevalence of 0.0045 [6]) was found to perfectly predict cases of schizophrenia or schizoaffective disorder in this genotype. These findings are also supported by a strong *MTHFR* 677 TT-dependent correlation between diagnosis and the vitamin B2/creatinine variable (*n*6, *rho* 0.828, *p* 0.042) and the equivalent ROC variable (*n*6 *rho* 1.000, *p* 0.000) (Supplementary Materials, Section S6). Other supportive findings for riboflavin conservation are the greater amount of riboflavin compared to the amount of metabolite implied in the Peak 2 area/Peak 1 area variable (*n*6, *rho* 0.886, *p* 0.019), with a Peak 2 area/Peak 1 area ROC correlate of *n*6, AUC 1.000, *p* 0.000, and similarly positive correlates for Peak 2/Peak1 amplitude. Taken together with the discussed literature on riboflavin excretion dynamics, these findings imply that the greater excretion of vitamin B2 by carriers of the *MTHFR* 677 TT genotype reflects higher vitamin B2 levels in plasma. Reduced riboflavin degradation in body or bacterial environments may contribute to this effect, and this dynamic is further discussed in Section 2.2.1 below.

#### 2.2.1. Vitamin B2 Excretion Levels Perfectly Predict Diagnosis for MTHFR 677 TT Genotype 

The notable ability of vitamin B2 and vitamin B2/creatinine excretion levels to predict a diagnosis of schizophrenia or schizoaffective disorder in the *MTHFR* 677 TT genotype can be attributed to several factors. Riboflavin (vitamin B2) is not synthesised in the body but is available in the diet and is also synthesised by microbiome bacteria [22,23,24]. After this, riboflavin is transported across the intestinal border and eventually excreted in the urine, along with the FMN and FAD co-analytes. However, in the setting of the relatively inactive MTHFR enzyme (coded by the *MTHFR* 677 TT genotype), there is an inadequate production of the major methylation metabolite 5-methyl tetrahydrofolate (5-MTHF), and this restricts methionine synthase (MS) activity and the upstream synthesis of methionine and its product S-adenosylmethionine (SAMe). Restricted MS activity holds back homocysteine conversion to methionine, along with homocysteine’s precursor S-adenosyl-homocysteine (SAH), which is itself the downstream product of SAMe. SAH accumulation then exerts competitive inhibition on creatine biosynthesis from glycine in a manner that restricts downstream creatinine synthesis [25]. Since 70% of SAMe-derived methyl groups are required for the methylation of guanidinoacetate (an intermediate substrate in the formation of creatinine’s precursor, creatine), the restriction of SAMe production also places a restriction on creatinine synthesis [26,27]. 

In this primary low methylation *MTHFR* 677 TT genotype-associated state, FAD dissociates away from the inactive MTHFR enzyme site, and it and its precursor flavoprotein, FMN, are readily available to cofactor other enzyme reactions [28]. For instance, FAD plays an indirect role in vitamin D activation to the 25-OH vitamin D [29,30] and FMN serves to activate Vitamin B6 to pyridoxal-5-phosphate (PLP) [31,32]. Moreover, FAD cofactors monoamine oxidase enzymes promoting the metabolism of indole-catecholamines and facilitating the activity of two key enzymes in the kynurenine pathway, which culminates in nicotinamide (NAD) formation [33,34,35]. In this setting, higher riboflavin Peak 2 excretion is thought to reflect increased FAD bioavailability due to FAD dissociation from the MTHFR enzyme. Thus, these two concurrent factors—released FMN, FAD, and restricted creatinine synthesis, combine to provide an explanation for the vitamin B2/creatinine biomarker and its ability to serve as a low methylation indicator and a case predictor for schizophrenia and schizoaffective disorder in the *MTHFR* 677 TT genotype. 

#### 2.2.2. Other MTHFR 677 TT-Dependent Biomarkers 

Other biomarkers discovered through ROC analysis together with odds ratio (OR) analysis results for the homozygous *MTHFR* 677 TT genotype are presented in Table 3 below. In this table, blue coded biomarkers can be attributed to the previously described low methylation state that is related to the relative inactivity of the MTHFR enzyme when coded by the *MTHFR* 677 TT genotype. This causes product 5-MTHF methyl donor insufficiency, which restricts methionine synthase (MS) ability to convert homocysteine to methionine in its usual manner. This restriction raises backed-up homocysteine levels [36] and constrains vitamin B12 cofactor utilisation by the MS enzyme. Consequently, homocysteine and vitamin B12 levels are raised (Table 3). To enable an alternative way for homocysteine to be converted to methionine, the zinc-dependent betaine homocysteine methyltransferase (BHMT) enzyme is activated. This enzyme serves a pathway across the methylation cycle, whereby homocysteine may be converted to methionine using trimethyl glycine (TMG) as an alternative methyl donor [37]. The compensative activation of this alternative pathway may set in motion a highly driven methylation state that allows S-adenosyl methionine (SAMe) to be once again synthesised from methionine. Unfortunately, zinc depletion by heightened BHMT activity is a side effect of that process. In this highly compensative methylation state, SAMe is readily available to facilitate adrenaline conversion from noradrenaline, explaining why low zinc levels and a high AD/NA ratio are the functional biomarkers of over-methylation, associated with the *MTHFR* 677 TT genotype [38].

#### 2.2.3. MTHFR 677 TT Genotype-Dependent Symptoms with High Dopamine, Adrenalin, and Elevated Vitamin B2

Case correlates for the *MTHFR* 677 TT genotype also reveal high DA ROC (*n*7, *rho* 1.000, *p* 0.000), which is attributed to plentiful vitamin B6 availability for dopamine synthesis by DOPA decarboxylase [40]. Other correlate signatures are attributed to SAMe availability for noradrenaline to adrenaline conversion (high AD/NA ROC: *n*7, *rho* 0.750, *p* 0.052). These high DA and AD signatures co-occur with 7 manic-flavoured symptoms (out of a possible 42 symptoms of psychosis), these being elated mood (*n*7, *rho* 0.986, *p* 0.000), motor-hyperactivity (*n*7, *rho* 0.986, *p* 0.000), thought preoccupation (*n*7, *rho* 0.956, *p* 0.000), distractibility (*n*7, *rho* 0.966, *p* 0.000), poor impulse control (*n*7, *rho* 0.956, *p* 0.001), and hostility (*n*6, *rho* 1.000, *p* 0.000). In this setting, there is an increase in symptom intensity rating (SIR), which reflects the total intensity rating for symptoms on a 1–7 scale. Moreover, such high-intensity symptoms relate to riboflavin (Peak 2 amplitude/Peak 1 amplitude (*n*6, *rho* 0.845, *p* 0.034). 

#### 2.2.4. In MTHFR 677 TT Carriers There May Be a Swing-Back to a Lower Methylation State with Depression

When the BHMT enzyme is harnessed to compensatively salvage SAMe production [37,41,42], SAMe facilitates the downstream formation of S-adenosyl homocysteine hydrolase enzyme (SAHH). This enzyme is responsible for converting S-adenosyl homocysteine (SAH) to homocysteine [43]. Some homocysteine product then passes into the transsulfuration pathway, where the enzyme cystathionine beta synthase (CBS) utilises vitamin B6 as a cofactor in the process of its metabolism [44]. Because vitamin B6 is readily activated by FMN it is available to activate the CBS enzyme and many other vitamin B6 co-factored body enzymes. For instance, vitamin B6 will facilitate the dopamine producing enzyme DOPA decarboxylase [40], which explains elevated dopamine levels and the elevated mood (manic) symptoms previously described [45]. At the same time, there is potential for FAD to be readily available for the MAO metabolism of indole-catecholamines, which depletes serotonin and catecholamine stores with potential for a sudden swing from manic symptoms into a deeply depressive state—a clinical complication that needs to be guarded against through frequent biomarker monitoring in carriers of the *MTHFR* 677 TT genotype. These functional understandings about the risk of depression in the carriers of the *MTHFR* 677 TT genotype may explain a recent meta-analysis report on the *MTHFR* C677T polymorphism (TT genotype) being associated with an increased risk not only of schizophrenia but also of severe depression in the general population [46].

### 2.3. Results and Discussion for the MTHFR 677 CC Genotype

#### 2.3.1. Biomarkers for the MTHFR 677 CC Genotype Are Characterised by Elevated Catecholamine and 5-HIAA Signatures 

ROC and odds ratio results for single and compound *MTHFR* 677 CC-dependent biomarkers are presented in Table 4 below, alongside the analysis of their theoretical significance. In contrast to the *MTHFR* 677 TT genotype results described above, these diagnostic biomarkers mainly represent low methylation biochemistry and are presented in blue. Methyl hydroxymandelic acid (MHMA) is a metabolite directly derived from the FAD facilitated metabolizing action of the monoamine oxidase enzyme (MAO-B) in a setting where SAMe facilitates the conversion of noradrenaline (NA) to adrenalin (AD) [47]. When there is insufficient availability of FAD catecholamine metabolism by MAO-B, catecholamines are conserved and their levels exceed the intermediate MHMA metabolite. Then, if SAMe is also unavailable to cofactor end-stage catecholamine metabolism through the enzyme catechol-o-methyltransferase (COMT), unmetabolized catecholamines further accumulate. Thus, the findings presented in Table 4 demonstrate ROC biomarkers of AD/MHMA and NA/MHMA, supported by the case–correlation signatures of NA/MHMA (*n*62, *rho* 0.322, *p* 0.011), NA/MHMA ROC (*n*62, *rho* 0.327, *p* 0.010), and NA + AD/MHMA (*n*62, *rho* 0.353, *p* 0.005). Interestingly, NA/MHMA also relates positively to case symptom intensity (SIR: *n*63, *rho* 0.452, *p* 0.000) and the extended duration of illness (DOI: *n*58, *rho* 0.554, *p* 0.000). 

#### 2.3.2. MTHFR 677 CC Genotype Is Characterised by Case Correlates for Low Activated Vitamin Biomarkers

This study has demonstrated predictive biomarkers for Peak1/Peak2 (Supplementary Materials, Section S9), where Peak 1 is presumed to be increased riboflavin degradation products and Peak 2 has been identified as riboflavin in the urine of *MTHFR* 677 CC genotype carriers. Reduced riboflavin bioavailability can in turn, lead to lower FMN and FAD synthesis, which then restricts the activation of vitamin B6 and vitamin D [29,31,48,49]. In this context, Table 4 demonstrates that low levels of activated vitamin B6, vitamin D, and folate are strong indicators of diagnosis in the *MTHFR* 677 CC genotype. This evidence is supported by negative case correlates for vitamin B6 levels (*n*63, *rho* −0.335, *p* 0.007), vitamin D levels (*n*64, *rho* −0.344, *p* 0.005), and folate levels (*n*57, *rho* −0.407, *p* 0.002), where Spearman’s correlation *p* < 0.05 is considered statistically significant and *p* < 0.01 is highly significant (Supplementary Materials, Section S7).

#### 2.3.3. Functional Interactions of Biomarker Findings for the MTHFR 677 CC Genotype 

Since FAD is a rate-limiting cofactor for the MTHFR enzyme, insufficient FAD synthesis from lower riboflavin will adversely affect the activity of this enzyme. Consequently, the low 5-MTHF product of the MTHFR enzyme is once again found to be the culprit for low methylation in the CC carrier state. Given that the thermolabile MTHFR enzyme in the TT genotype also has low 5-MTHF output, this outcome achieves little that can differentiate between the TT and CC genotypes. However, what is different is that the *MTHFR* 677 CC-coded enzyme has normal activity but is simply restricted by a lack of riboflavin supply to synthesize its required FAD cofactor. In both cases, restricted 5-MTHF production will still render methionine synthase (MS) partially inactive, whilst its ancillary vitamin B12 cofactor and precursor homocysteine may accumulate, displaying a trend towards higher rather than lower levels (as indicated within the compound biomarkers of Table 4).

5-Hydroxyindoleacetic acid (5-HIAA) is serotonin’s main metabolite, and serotonin’s precursor is L tryptophan. FAD and vitamin B6 are utilised in the kynurenine pathway, which is an alternative pathway for the L tryptophan metabolism. Since the principal enzyme tryptophan pyrrolase in this pathway lacks activation without vitamin B6 and FAD [33,50], L tryptophan is conserved and directed towards serotonin synthesis and metabolism. Consequently, there is overflow of serotonin metabolite (5-HIAA) into the urine [33,51], which explains the significant finding of the 5-HIAA correlate (*n*65, *rho* 0.263, *p* 0.000) for the *MTHFR* 677 CC genotype.

The high serum histamine ROC result within Table 4 is notable. High histamine has been used by natural health practitioners as a biomarker indicative of low methylation status [52]. This occurs because SAMe is required as a cofactor for an enzyme undertaking histamine metabolism. Empirical evidence of histamine’s role in schizophrenia has been largely restricted to case studies. However, given the appearance of a high histamine ROC in this study and its association with markers representing the *MTHFR* 677 CC-dependent low methylation phenotype, we at last, have firm evidence to justify the use of high histamine levels as a biomarker for low methylation status.

Copper is utilised in the conversion of dopamine (DA) to noradrenaline (NA) by the copper containing enzyme, dopamine-beta-hydroxylase (DBH) [53]. This enzyme may find its activity stalled in a setting of low methylation, where SAMe unavailability restricts noradrenaline conversion to adrenalin (downstream of dopamine synthesis) and restricts catecholamine synthesis by COMT (Scheme 1). The reciprocal relationship that exists between serum and plasma copper (Cu) and zinc (Zn) [39] thus explains the finding of elevated % free Cu/Zinc ratio (Table 4). So, it seems that an elevated % free Cu/Zinc ratio is unable to differentiate between the *MTHFR* 677 TT and CC genotypes; however, there is a functional differentiation. The functional dynamic in the *MTHFR* 677 TT genotype is the excess utilisation of zinc by the BHMT enzyme, whereas in the *MTHFR* 677 CC genotype, it is explained by copper release from the stalled DBH enzyme. In either context, it is notable that free copper acts to inhibit the cystathionine beta synthase (CBS) enzyme [54], thereby limiting homocysteine metabolism via the transsulfuration pathway. In the *MTHFR* 677 CC genotype, there is the additional factor of low vitamin B6 bioavailability, which limits cystathione-beta-synthase (CBS) activity, thereby limiting glutathione (GSH) synthesis at the end of this pathway. Also, free copper inhibits this enzyme’s activity, allowing the combined effects of high copper and low vitamin B6 (PLP) to restrict the production of glutathione at the bottom of this pathway. Then, lack of FAD (and the reduced phosphorylated form of Nicotinamide (NAD), NADPH) further restricts glutathione availability by restricting cofactor availability for the glutathione reductase enzyme to convert oxidised glutathione (GSSH) back to its reduced activated (GSH) form [55].

Once glutathione is produced, it interacts with copper and zinc, binding superoxide dismutase (SOD), which is a powerful antioxidant enzyme. SOD’s binding of zinc may restrict zinc availability and contribute to the low zinc, higher % free Cu dynamic (% free Cu/Zn) that is detected in Table 4 as a biomarker related to the *MTHFR* 677 CC genotype. Using this zinc-binding mechanism, a brake may be placed on the ability of the BHMT enzyme to drive any compensative shift to a high methylation state. In this setting, the glutathione peroxidase enzyme uses glutathione to break down hydrogen peroxide generated by SOD antioxidant activity [56]. However, with restricted GSH availability, hydrogen peroxide accumulates with accompanying potential for free radical tissue damage. Such oxidative stress has a known relationship with schizophrenia [57], and in this context it is notable that in this study, we discovered that the *MTHFR* CC genotype has a strong relationship with a reported history of developmental delay (*n*62, OR 106.6, *p* 0.000), which may explain why oxidative stress is reported in autism [58]. Since less glutathione is available to assist SOD’s antioxidant defence against free radical accumulation, heme oxidase (HO) may be active (Scheme 1), and it is conjectured that hydroxyhaemopyrroline-2-one (HPL) is released into the urine as the HO oxidative degradation product of haeme porphyrin. High levels of haeme may also be released from the haeme containing enzyme tryptophan pyrrolase at the top of the kynurenine pathway. During inflammation, this enzyme may be primed by inflammatory factor NFKappa b (NF-Kb) but may release its haeme for metabolism, as it has its activity suspended in an environment lacking FAD and vitamin B2. Such inflammatory induction of NF-Kb has a known relationship with schizophrenia [59]. We have not reported on levels of this HPL molecule in this study and will do so in a future publication. High levels of this molecule leads to metabolic crisis in mitochondria [60]. High levels of HPL have been detected in the urine of patients with schizophrenia who do not possess the homozygous *MTHFR* 677 polymorphism [61], and significant correlates for heightened HPL have also been linked to multiple symptoms of schizophrenia [15,45,62,63].

By aligning our biomarker results in this study with existing research literature evidence, it seems that carriers of the *MTHFR* 677 CC genotype have potential for a “perfect storm” of gastrointestinal inflammation, oxidative stress, nutrient malabsorption, and riboflavin inhibitory degradation factors that can conspire to bring about the low absorption of vitamin nutrients and low body riboflavin.

#### 2.3.4. ROC Correlate Biomarkers for the MTHFR 677 CC Genotype

ROC results, with significant *MTHFR* 677 CC-dependent correlates (Supplementary Materials, Section S8), were for:

- elevated % free copper to zinc ratio ROC (*n*59, OR 9.9, *p* 0.052);
- elevated AD/MHMA ROC (*n*59, OR 25.4, *p* 0.0001);
- elevated vitamin B12/vitamin D ROC (*n*59, OR 21.29, *p* 0.011);
- low vitamin B6 ROC (*n*59, OR 12.1, *p* 0.015).
  - where Spearman’s correlation *p* < 0.05 is considered statistically significant and *p* < 0.01 is highly significant. Then, using cross tab analysis and logistic regression (Supplementary Materials, Section S9 and Table 5), based upon the prevalence of schizophrenia in the general Australian population (0.45%) [6], a predictive biomarker model was obtained for diagnosis, with 93.3% sensitivity and 92.6% specificity and a negative predictive value (NPV) of 100%. This high percent NPV value means that the model has excellent screening potential for ruling out schizophrenia disease in the general population. Other predictive biomarkers identified by *MTHFR* 677 CC-dependent ROC correlative results were for low vitamin B6, AD + NA/MHMA, and 5-HIAA, which confirms our previous ROC findings shown in Table 4. Taken together, these correlative findings indicate that low vitamin B2 and vitamin B6, along with elevated catecholamines and serotonin excretion product (5-HIAA) are key molecular biomarkers for psychosis in carriers of the *MTHFR* 677 CC genotype. The significance of elevated vitamin B12/vitamin D ROC as an indicator of a low methylation state is further discussed in Table 4 and Section 2.3.4 below. These findings provide a functional understanding for low methylation states in the *MTHFR* 677 CC genotype. When compared with biomarkers of high compensative methylation observed in the *MTHFR* 677 TT genotype, the high vitamin B12/vitamin D blood biomarkers occurring alongside urinary biomarkers of conserved catecholamine and 5-HIAA have the potential to identify a low methylation state in any *MTHFR* 677 CC carrier who has unclear or limited symptoms. Furthermore, this certainty can be obtained purely from entering these results directly into an algorithm constructed to predict this biochemical phenotype.

### 2.4. Results and Discussion for the MTHFR 677 CT Genotype

#### Biomarkers for the Heterozygous MTHFR 677 CT Genotype

Table 6 presents ROC and odds ratio characteristics for variables within the *MTHFR* 677 CT genotype. Due to the heterozygous nature of alleles within this genotype, it was expected that carriers might inherit biomarkers demonstrating the overlap, reinforcement, or cancellation of biomarker effects. These expectations were met by ROC, logistic regression, and correlation analysis findings, showing simultaneous high and low methylation phenotypes.

As expected, for this mixed, *MTHFR* 677 CT genotype, many of the biomarkers identified have overlap with those of the *MTHFR* 677 CC genotype, which makes it difficult to differentiate any one biomarker that is distinctive for carriers of the *MTHFR* 677 CT genotype. On comparison of biomarkers in Table 4 and Table 5, compound biomarkers DA × 5-HIAA ROC and NA + AD/MHMA would seem to offer best differentiation ability for case *MTHFR* 677 CT genotype prediction and monitoring purposes.

The above results were supported in terms of case correlation by the following findings:

- Low methylation markers are present for conserved catecholamines (high DA: *n*61, *rho* 0.384, *p* 0.002, high NA: *n*61, *rho* 0.686, *p* 0.000, NA/MHMA: *n*60, *rho* 0.545, *p* 0.000 and AD/MHMA: *n*60, *rho* 0.427, *p* 0.001). Also present are markers where riboflavin metabolites exceed riboflavin excretion (Peak 1–Peak 2 area ROC: *n*54, *rho* 0.316, *p* 0.020), with a marker representing low activated folate (low folate ROC: *n*62, *rho* 0.323, *p* 0.011).
- In the correlative data set, there is also some evidence of a shift to a compensative high methylation state, with the AD/NA ROC signature implying plentiful SAMe (*n*61, *rho* 0.339, *p* 0.007). This is accompanied by an HPLC riboflavin signature for case-ness (area under Peak 2 ROC: *n*54, *rho* 0.382, *p* 0.004). Finally, in the correlative data, we then find confirmatory evidence of a strong relationship between vitamin B2 ug/L levels and activated vitamin B6 (PLP) levels (*n*53, *rho* 0.449, *p* 0.001).
- The expectation of mixed biomarkers representing the overlapping effects of T and C alleles was clearly met by the data identification of a range of compound biomarkers, featuring a predominance of unutilised, trapped vitamin B12 and homocysteine, with low, overutilized zinc. These markers include elevated vitamin B12 ROC (*n*62, *rho* 0.228, *p* 0.075), vitamin B12/zinc × folate (*n*58, *rho* 0.280, *p* 0.027) and elevated [vitamin B12 × % free copper]/[zinc × folate] (*n*62, *rho* 0.277, *p* 0.029); [vitamin B12 × % free Cu × homo-cysteine]/[zinc × folate × vitamin B6] (*n*58, *rho* 0.294, *p* 0.025); and/or [vitamin B12 × % free Cu × homocysteine]/[zinc × folate × vitamin B6 × vitamin D] (*n*57, *rho* 0.277, *p* 0.037). Similar mixed results were found via logistic regression analysis, where color-coded results are reported below, at a 95% confidence level.
- Vitamin D/vitamin B12 ROC (*n*58, OR 228.28, *z* 1.85 *Pz* 0.065);
- high NA/MHMA ROC (*n*58, OR 5.63, *z* 1.85, *Pz* 0.064);
- high AD/MHMA ROC (*n*58, OR 17.86, *z* 1.93 *Pz* 0.054);
- low folate ROC (*n*58, 28.8, *z* 2.72, *Pz* 0.007);
- Histamine + NA (*n*58, OR 1.17, *z* 1.95, *Pz* 0.018).

In the above list, *z* is coefficient of predictor variables/standard error (Std. Err.), where OR (Odds Ratio) is exponential of the *z* coefficient and *Pz* is the probability associated with the *z* factor, at a 95% confidence level.

Elevated histamine is indicated in these results and can be expected to occur in a low methylation setting where SAMe is unavailable for histamine metabolism by the histamine methyltransferase (HMT) enzyme. Also, vitamin B6 is unavailable for histamine metabolism by diamine oxide (DAO) [64]. As explained earlier, elevated NA is attributed to its inhibited metabolism by MAO-B, in a low methylation, low vitamin setting, which is compounded by insufficient SAMe cofactor for the conversion of NA to AD [38,45].

Carriers of this heterozygous *MTHFR* 677 CT genotype demonstrated a full hand of symptoms, noted to be highly contrasting in flavour, possibly representative of a mixed affective state or a rapid cycling affective process. Also, carriers of this *MTHFR* 677 CT genotype experience the maximal number (40 out of 42) of significant case-correlated symptoms (Supplementary Materials, Section S10), and multiple adverse outcomes that will be presented and discussed in a later publication. This potential for mood destabilisation through biochemical effects derived from overlapping T and C alleles deserves further research attention. Indeed, there is some evidence for differential brain methylation states in the 2014 findings of Xiao et al., who reported different DNA methylation levels between frontal and anterior cingulate brain regions in schizophrenia and bipolar subjects [65]. Further findings by Rizzardi et al. in 2021 also reported DNA methylation heterogeneity in the brain associated with schizophrenia [66]. 

### 2.5. Results and Discussion of Molecular and Trace Element Biomarkers across All MTHFR C677T Genotypes

**Figure 3:**
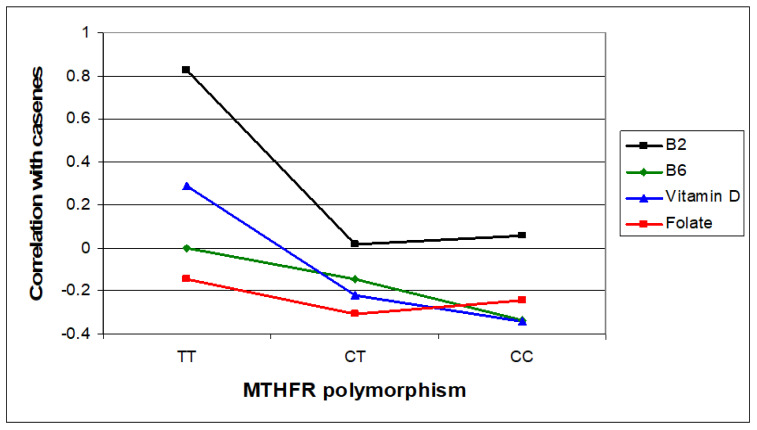
Relative case correlation strengths for vitamin B2, B6, and D and folate across *MTHFR* C677T genotypes.

**Figure 4:**
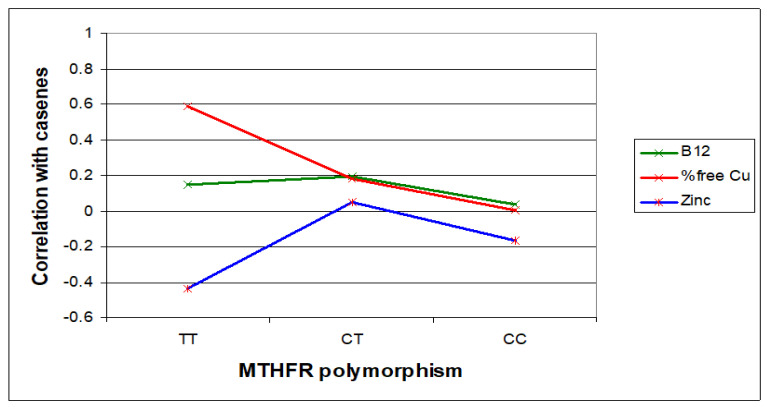
Case correlative strength for B12 and zinc within the TT, compared with CC genotype.

**Figure 5:**
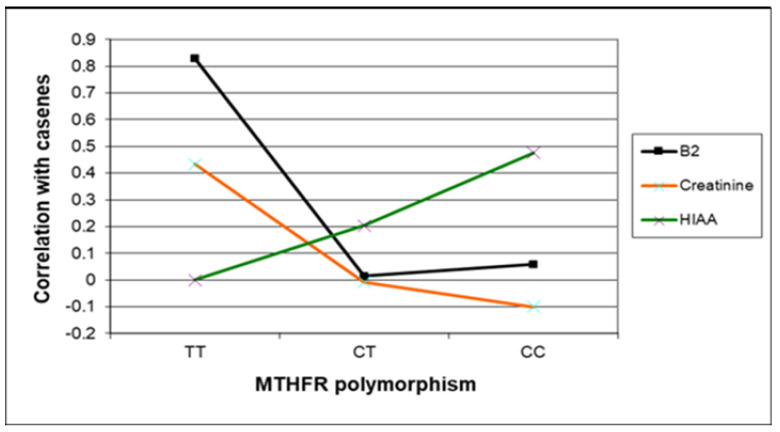
Case correlative strength for B2, creatinine, and 5HIAA within the TT, compared with CC and CT *MTHFR* 677 genotypes.

In this section, Figure 3, Figure 4 and Figure 5 present the major biomarker correlates across *MTHFR* C677T genotypes. Table 7 summarises correlative findings for biomarkers across all *MTHFR C*677T genotypes, according to low or high methylation (with further cross genotype data presented in the Supplementary Materials, Sections S6, S7, and S9). Table 8 then provides a final summary of all biomarker findings across the three *MTHFR* C677T genotypes with functional explanations and symptom affiliations.

## 3. Future Research Directions

A major drawback in the management of psychoses, such as schizophrenia and schizoaffective disorder has been the lack of biomarkers to aid in the diagnosis and treatment of patients with these disorders. Also, there are currently few proposals for subtyping psychotic psychiatric conditions such as schizophrenia and schizoaffective disorder [67]. The results of this research suggest that methylation-based classification may prove to be a meaningful way forward for subtyping various forms of psychosis.

This research study finds that *MTHFR* C677T genotypes are associated with different functional methylation phenotypes with cross genotype trends in biomarker levels of catecholamine, vitamins, and other intermediate substances. Our previous research [61] has also demonstrated that such changes are related to symptoms of psychosis and abnormal mood and there is ample incentive for further research to test the biomarker efficacy according to therapeutic applications. 

Since riboflavin’s derivative FAD is the cofactor for MTHFR enzyme, riboflavin can be considered to play a remote, though important role in supporting this enzyme to supply 5-MTHF for replenishing the methylation cycle and synthesizing SAMe for DNA methylation. Therefore, the findings of this study have meaning and translational scope for DNA methylation and the expression of many other genes [68,69,70]. SAMe’s methyl groups are also required for the liver synthesis of many biologically active compounds, including phosphatidyl choline and sphingomyelin [71], and a lack of these substances has been associated with the demyelination of the brain in schizophrenia [72,73].

Future research will seek to validate current results and link *MTHFR* C677T genotype-related functional phenotypes to DNA methylation and define methylation-related phenotypes across a wider range of genetic expression. We will also broaden the scope of the current work through phenotype refinement towards methylation classification and harness algorithmic and machine learning applications translation across different populations. 

## 4. General Therapeutic Applications 

It is important to recognize that *MTHFR* C677T gene testing alone cannot form a basis for the treatment of schizophrenia or schizoaffective disorder, as functional methylation in biochemistry is complex and governed by compensatory pathways and feedback mechanisms. These processes can create flux under conditions of biochemical change brought about by nutritional and genetic factors, medications, environmental toxins, stress, or other forms of pathology [74]. We have described the dynamics in these flux processes in detail in a previous review paper that outlines knowledge gaps where further research is required [15].

In this study, we have shown that *MTHFR* C677T genotypes are accompanied by biochemical phenotypes representing functional low and high methylation states. These states are, in turn, characterised by different riboflavin dynamics with different indole-catecholamine metabolism, vitamin activity, and metabolism of homocysteine and histamine. These findings point towards the genotype–phenotype-dependent adjunctive management of psychosis symptoms with precision treatment to dial methylation status up or down, according to the results of blood and urine biomarker tests. This requires careful monitoring and judicious decision making, so the ideal, least invasive option, is to use dietary methods for achieving this objective.

Since the majority of participants in any sample population will be *MTHFR* 677 CC or CT carriers, it is understandable that schizophrenia and other psychiatric illnesses have been found to be related to riboflavin (vitamin B2) and pyridoxine (vitamin B6) deficiency [75] and to also be linked to urban populations [76]. In cities, supplies of riboflavin in the form of fresh meat, milk, legumes, and green vegetables are less readily available. Riboflavin deficiency has also been reported in aged populations [77], where poor dietary sources and age-related achlorhydria [78] contribute to limited riboflavin availability and absorption. Riboflavin levels are high in baker’s or brewer’s yeast in bread and beer, respectively [79], and it may be that carbohydrate and beer addiction relate to riboflavin-seeking behaviour [80]. 

Agents with capacity to inhibit methylation such as SAH [81,82], DNA methyltransferase inhibitors, and histone deacetylase inhibitors have been considered for treating high methylation states [83,84], which may also be present in cancer [49,85]. However, changing DNA or histone methylation levels do not necessarily change the methylation status of the underlying biochemistry [86,87].

A starting point for enhancing low methylation states is lifestyle changes to ensure an environment free of environmental toxins, smoking fumes, and excessive stress [88]. There is also the need for the exclusion of pathology and the treatment of chronic infection, inflammatory or immune challenges, which may induce an over methylation stress response that depletes indole-catecholamine neurotransmitter levels. The alteration of existing medication may be necessary as oral contraceptives, proton pump inhibitors, nitrous oxide, ibuprofen, some antibiotics, metformin and aspirin, can inhibit methyltransferases or alter the microbiome in a manner that suppresses methylation [89,90]. Ensuring the adequate dietary intake of methionine, cysteine, taurine, riboflavin, niacin, pyridoxine, betaine, and choline, along with the adequate intake of sulphur, zinc, magnesium, and potassium, should also be considered. Supplementing with SAMe synthesis supporting agents such as methyl folate, methyl cobalamin (methylated vitamin B12), vitamin B6, and trimethyl glycine are further therapeutic options in the low methylation setting. However, regular monitoring must be undertaken to offset the risk of over-supplementation setting in motion opposing feedback mechanisms. DNA methyltransferase 1 (DNMT1), activating compounds such as resveratrol, genistein, curcumin, and other natural polyphenols that stimulate the activity of DNA methyl transferases, have been shown to be effective for rectifying under methylation in some cell types, but these have yet to be trialled for schizophrenia [91]. There is also scope to consider probiotic therapy to boost microbiome riboflavin or folate production. Many *bifidobacteria* synthesize folate [92], and other microbiome inhabitants such as *Bacillus subtilis* [23,93] or *Lactobacillus fermentum* [22] that synthesize riboflavin may emerge as important ways of modulating activity of methylation pathways. However, the challenge of probiotic use is their durability within the gut microbiome and their prevailing efficacy despite change in the biochemical environment. Targeted probiotic treatment trials that take methylation genotypes and phenotypes into account are therefore urgently required.

Since methyl groups are added by SAMe to DNA bases (usually cytosines), the future of methylation suppression may lie in the proofreading of DNA and the mismatch repair of bases with added methyl groups. There are mechanisms that can detect and fix damaged DNA that can impair DNA methyltransferases [94] or use small interfering RNA (siRNA) to recruit DNA methyltransferases for the induction of methylation-related silencing [95]. Future therapy options also include gene transfer therapy, the pharmacological modulation of gene expression, and DNA protein replacement or enhancement [96]. There is of course, the underlying caveat that all these therapy options be accompanied by carrier genotype screening, pre-symptomatic diagnosis, and genetic counselling.

## 5. Summary of Genotype—Phenotype Specific Findings with Therapeutic Implications

In this study, the 5.2%-prevalent homozygous *MTHFR* 677 TT polymorphism demonstrated diagnosis-predictive biomarkers that are mainly related to the incapacity of the disabled MTHFR enzyme. In this setting, the MTHFR enzyme cannot utilize FAD and dissociates from the enzyme. Thus, FMN and riboflavin precursor substances may be utilised elsewhere in the body biochemistry (Scheme 1). In this TT genotype context, our research findings reflected this theory by showing higher tissue levels of riboflavin (vitamin B2), FMN, and FAD, and it was notable that the vitamin B2/creatinine biomarker was able to detect schizophrenia or schizoaffective disorder in the small TT genotype sample, with 100% sensitivity and 100% specificity. Other diagnosis-predictive biomarkers of low folate and elevated vitamin B12 and homocysteine are explained by the enzymes’ inability to produce the primary methylation product 5-MTHF. In this low methylation state, the methionine synthase (MS) enzyme lacks its 5-MTHF cofactor and is unable convert homocysteine to methionine via its usual route, so homocysteine accumulates behind it. Homocysteine is a known risk factor for cardiovascular disease, Alzheimer’s disease, depression, and many other pathologies [97]. However, we also found biomarkers indicative of a high methylation state. This state is reasoned to occur to offset low methylation and produce methionine (and thus, SAMe) via an alternative route. This route is available via the BHMT enzyme, which may overcompensate in salvaging SAMe production, leading to a high methylation state. This functional state is characterised by low zinc from the over-utilisation of BHMT’s zinc cofactor (present in low zinc correlates and in the form of a high % copper to zinc ratio) and high AD/NA ratio, representing SAMe availability for conversion of NA to AD. Due to SAMe’s co-factoring of the COMT enzyme and FAD’s dissociative release from the disabled MTHFR enzyme, there is plentiful FAD supply for co-factoring the MAO-B enzyme, enabling the limited metabolism of catecholamines, which produces toxic catechol–aldehyde substances with neurotoxic potential related to tau pathology and damage to the noradrenaline-producing locus coeruleus [98,99]. However, after a switch to a higher compensative methylation state has occurred, plentiful SAMe is available to convert noradrenalin to adrenaline and facilitate end-stage catecholamine metabolism by the COMT enzyme. Thus, the combination of the FAD-facilitated MAO metabolism and the SAMe-facilitated COMT metabolism has the potential to bring about a rapid, severe drop in catecholamine levels. In this study, manic-flavoured symptoms associated with dopamine retention (from low SAMe) occur alongside depression symptoms that are reminiscent of a manic depression (bipolar affective) state. Taken together, these diagnosis-predictive biomarkers imply that the judicious monitoring of urine for both 5-HIAA and catecholamine levels should be carried out for all TT carriers. If urinary catecholamine levels are found to be low or AD/NA levels high (indicative of a shift to high methylation), then consideration can be given to supplementation with 5-MTHF. This supplementation has potential to restore methylation via the usual route and offset the need for a switch to high compensative methylation state. However, if these biomarkers indicate that a switch has already taken place, the potential for exacerbating depression by any ongoing treatment with dopamine receptor blocking medication requires clinical consideration. In this setting, frequent biomarker monitoring may assist clinical decision making and may guard against the development of distressing Parkinsonian symptoms in carriers of the *MTHFR* 677 TT genotype. 

The 48.5% prevalent *MTHFR* 677 CC genotype demonstrated 93% sensitivity and 92% specificity and 100% negative predictive value—based upon biochemical variables alone. This predictive model included lower vitamin B6 and lower folate cofactor levels, high catecholamine excretion levels, a higher serotonin excretion product (5-HIAA), high histamine levels and HPLC findings suggesting higher vitamin B12 degradation products and lower vitamin B2 levels. Within this genotype, biomarkers suggesting low methylation status were negative correlates, ROCs, and predictor variables for activated vitamins B6, vitamin D, and folate alongside positive indices for high histamine, vitamin B12, unmetabolized catecholamines, and a noradrenaline to adrenaline ratio. Against these dynamics, the valence of higher vitamin B12 to lower vitamin D (expressed as vitamin B12/vitamin D) emerged as a very useful biomarker for confirming a prevailing low methylation state. These findings indicate potential for either dietary, probiotic or supplement boosting of vitamin B2, vitamin B6, vitamin B3 (niacin), and vitamin D, along with the consideration of low histamine diet or antihistamine use if histamine levels are exceedingly high. Since low methylation conditions predominate in this genotype, well-monitored methyl group boosting agents, such as choline, betaine, methionine, or methylated folate or methylation boosting cofactors such as methylated vitamin B12, vitamin B6, magnesium, zinc, and sulphur may play an adjunctive treatment role [100]. High histamine is confirmed as a notable biomarker in this low methylation associated genotype. Histamine and vitamin B2 are produced in the gastrointestinal system and high histamine may contribute to gastrointestinal inflammation [101] in a setting where such inflammation also has a recognised association with vitamin B2 (riboflavin) deficiency [102,103]. This inflammatory setting may jeopardize the vitamin absorption of other nutrients, such as folate, vitamin B6 (PLP), and even riboflavin itself. The microbiome may also be altered in a manner that diminishes the bacterial production of these vitamins [104]. For these reasons, therapeutic attention to gastrointestinal function is an important consideration in carriers of the *MTHFR* 677 CC genotype and, if gastrointestinal inflammation is detected, treatment with anti-inflammatory probiotics such as *lactobacillus* and *bifidobacteria* species [105] may be warranted, alongside a low histamine diet and targeted vitamin B2 supplementation or dietary adjustment.

The 46.3%-prevalent CT genotype achieved similar predictive strength for a mixed methylation phenotype in schizophrenia and schizoaffective disorder, and MTHFR 677 CT genotype-related biomarkers demonstrated considerable overlap with those of the MTHFR 677 CC genotype. Within this genotype, biomarkers with best potential for demonstrating mixed components of methylation were positive indices for dopamine X 5 hydroxy indoleacetic acid and NA + AD/MHMA. The low methylation marker of higher vitamin B12 to lower vitamin D (expressed as vitamin B12/vitamin D) emerged again as a very useful biomarker for confirming a prevailing low methylation state in this CT genotype. Carriers of this genotype were characterised by contrasting symptomatology that may possibly represent a mixed or rapid cycling affective state, so the consideration of mood-stabilizing medications may be a treatment option (although sodium valproate should be used with due consideration as it may interfere with DNA methylation [106] and may raise homocysteine levels and lower folic acid levels [107]). A natural solution is to monitor biomarkers and use flavin-synthesizing probiotics and methyl donors, such as choline, methionine, betaine, methylated B12, vitamin B6, and methylation adaptogens such as curcumin and quercetin provide subtle, yet effective, methylation support.

## 6. Materials and Methods

Case and control participants were approached for recruitment in ward and community clinic settings. Sites of participant assessment were psychiatry clinics in the community catchment area of the Western Adelaide and Basil Hetzel Institute for Translational Health and the Queen Elizabeth Hospital at Woodville, South Australia. This area consisted of 22,000 persons. Multi-ethnic participants diagnosed with well-defined first episode psychosis or established schizophrenia or schizoaffective disorder between the age of 18 to 60 years were recruited over the three years of this study. Of 89 consenting cases, 7 did not pass the assessment and 15 were excluded because they were receiving serotonin re-uptake inhibitors (SSRIs) or serotonin-noradrenalin re-uptake inhibitors (SNRIs), which conserve indole-catecholamines and were therefore considered to have the potential to alter indole–catecholamine excretion levels. Control participants were drawn from a catchment area in northwest Adelaide via a sample of 2487 persons associated with a general population data set. On the further clinical assessment of 72 recruited individuals, only 67 met the full project inclusion criteria, so data analysis was conducted on 67 participants and 67 controls, which included 63 pairs for gender and age (within 5 years). In a case–control study, the pairing of data for analysis is legitimate and may be more efficient than a matched analysis because matching can introduce bias, as matched variables may also match risk factors for exposure [108]. This may be the case when mental illness disorders share a range of risk factors and occur as part of a spectrum, as is likely for schizophrenia. *MTHFR* C677T-dependent data characteristics are presented in the Supplementary Materials, Section S5. All participants gave informed written consent and the study conformed to principles of the Declaration of Helsinki. Methods and protocols conformed to the regulatory standards of the Queen Elizabeth Hospital Research Ethics Committee (ethics approval no: 2009139). 

Both case and control participants were subject to similar exclusion criteria and all participants were rated for clinical and subclinical symptoms, even though no control participants met diagnostic criteria for mental illness. Participant inclusion and exclusion criteria for this study are presented in the Supplementary Materials, Sections S1 and S2. Multiple exclusion criteria were imposed upon all study participants to minimize confounding variables and to strip psychosis down to its core condition. This approach meant excluding participants who were substance abusing or who had been investigated for organic causes of psychosis or who were experiencing extra-pyramidal side effects of medication [109]. Also excluded were participants with any form of motor disability; perceived, documented, or overt visual disability or sensory/conductive hearing loss; or any neurophysiological condition, as these conditions were capable of confounding outcome measures on visual or auditory perception or processing assessments. Participant medication exclusion criteria were designed to reduce factors likely to contribute to the unstable levels of candidate markers. Therefore, patients under treatment with Clozaril, Olanzapine, antihistamines, or vitamin or mineral supplements were excluded. In this manner, highly characterised cases of functional psychosis were recruited.

Biological samples were taken prior to diagnostic assessment by psychiatrically trained assessors who were blind to laboratory results. Antipsychotic medication was unchanged during the assessment period and to minimize severity bias, hospital cases were assessed close to their point of discharge into the community. Participant interviews collected demographic and medical history data that included age, duration of illness, history of developmental delay, accessory diagnoses, medication, and medical comorbidity. Also assessed were a range of symptoms and risk cofactors that will be discussed in more detail in later publications. Rating measure details are presented in the Supplementary Materials, Section S3. Diagnoses were based upon the International Diagnostic and Statistical Manual of Mental Disorders, Fourth Edition (DSM IV-R) [110], and symptoms were check-listed against this standard. Participants were also rated using the Brief Psychiatric Rating Scale (BPRS) [111]. The BPRS also included additional items from the Positive and Negative Syndrome Scale for Schizophrenia (PANSS) [112]. A total of 42 symptoms were rated for each participant and each symptom rated for intensity (1–7), allowing a total Symptom Intensity Rating (SIR) to be constructed.

Vitamin cofactor and trace element candidate markers for the study were selected for their accessibility on commercial testing as well as their role as cofactors in the biochemistry pathways of interest. Laboratory assays were carried out for a range of analytes, with methods depicted in the Supplementary Materials, Section S4. Fasting blood and urine samples were collected between 9 and 11 am daily. Blood samples were transported directly to the laboratory for analysis with no storage and urine samples frozen prior to transport. Serum samples were collected for *MTHFR* Ala222Val (C677T) methyl tetrahydrofolate reductase polymorphism and activated forms of vitamin D (25-OH), vitamin B12, vitamin B6 (PLP coenzyme form), copper, ceruloplasmin, and histamine. Plasma samples were collected for homocysteine and red cell zinc. From serum ceruloplasmin and plasma red cell zinc percentage free copper/red cell zinc was calculated via an equation. HPLC laboratory urine analysis was conducted for levels of creatinine, dopamine (DA), adrenaline (AD), noradrenaline (NA), and their metabolites (homovanillic acid (HVA) and methoxy-hydroxymandelic acid (MHMA)). The serotonin metabolite 5-hydroxyindoleacetic acid (5-HIAA) and riboflavin (vitamin B2) were also assayed from urine. Vitamin B2 (riboflavin) levels in urine are linearly related to tissue vitamin B2 levels [19,113]. Riboflavin analysis was performed using high-performance liquid chromatography (HPLC). Riboflavin levels (μg/L) were directly assayed and two HPLC elution peaks were identified. The amplitude and area under these peaks were measured and these HPLC findings are shown in Figure 1 and Figure 2. Riboflavin levels (μg/L) calculated from the amplitude of Peak 2 corresponded to those of the standard riboflavin stock solution, and details are presented in the Supplementary Materials, Section S4. FAD and FMN standard solutions (Sigma-Aldrich Inc., St. Louis, MO, USA) were then used to determine the position of these flavoproteins with respect to Riboflavin (Peak 2) elution (Supplementary Materials, Section S4) [17,18,19,21,114].

Statistical analysis was performed for the three *MTHFR* C677T genotypes using parallel methods and a combination of parametric and non-parametric methods with *p* values of <0.05 considered statistically significant. Primary demographic analysis established means for sample characteristics with a 95% confidence interval, using SPSS (Version 20.0) statistical software package [115]. Later analyses used the Kruskal–Wallace H test [16] (Table 1), with other data characteristics supplied in the Supplementary Materials, Section S5. Receiver operating characteristic (ROC) curves to establish biomarker status for single and compound variables were determined using Stata statistics software, version 15.1 [116]. When an outcome variable had outstanding ability to discriminate between case and control outcome measures, the area under the ROC curve (AUC) was >0.9. When discrimination was excellent, it was 0.8–0.9. A curve with an area under curve of 0.7–0.8 indicates acceptable discrimination, and an area under the curve of 0.5 to 0.7 represents poor diagnostic discrimination [117]. In the odds ratio (OR), the diagnosis association data were also determined for some ROC variables, where OR ≥ 2 was considered important. For each *MTHFR* C677T genotype, Spearman’s correlation analysis was carried out on raw variables and promising markers derived from receiver operating characteristics (ROC). Spearman’s correlation *p* < 0.05 is considered statistically significant and *p* < 0.01 is highly significant. Spearman’s correlation analysis used the SPSS (Version 20.0) statistical software package [115]. The Benjamini–Hochberg procedure [118] was utilised to correct ROC < odds ratio and Spearman’s correlation results for the effect of multiple comparisons. Linear and logistic regression analyses were then conducted on promising variables identified from ROC, odds ratio, and Spearman’s analysis. Back-up cross-tab analysis was used when prediction was found to be 100% via these methods.

Logistic regression analysis was used for the biomarker prediction of diagnosis using identified ROC and correlation biomarkers that were dependent upon the *MTHFR* C677T genotype. Predictive models so derived also provided parameters of odds ratio for predictive variables within the models. The sensitivity and specificity of models were also provided in a setting where the sensitivity and specificity of predictive models were considered acceptable at ≥85 percent and ideal at ≥90 percent. A model with >90% predictive sensitivity has utility as a diagnostic predictor because it can predict >90% of cases of schizophrenia or schizoaffective disorder. In contrast, a >90% specificity model has utility for predicting whether an individual has schizophrenia/psychosis or not. The coefficient of predictor variables can be positive or negative, and odds ratios so estimated are the exponentials of the coefficient. The z value is the coefficient divided by the standard error of the coefficient. These methods of regression analysis allow the entry of raw laboratory results or easily transformed results into any algorithm formed from any strong predictive model. The large data set relating to this publication is still under embargo awaiting the publication of closely related findings. Supporting data specific to this publication are available in the Supplementary Materials and data set information is available from the corresponding author upon reasonable request and confidentiality conditions.

## 7. Limitations

Data gathered from participants in this study were gained in a real-world setting where symptoms of psychosis are sometimes obvious; therefore, assessors were not blind to participant status. Small sample size particularly affected the number of lower prevalence *MTHFR* 677 TT carriers that could be analysed in the final sample (*n*7, prevalence 5.2%). Despite this limitation, the TT genotype produced strong correlates for biomedical measures and sample numbers for this genotype lie within the prevalence range for world-wide populations where they meet the lower range for the Australian population [118]. Borderline power may mean that positive results for variables explored in this study are likely to be correct at the significance level stated, but some relationships that do exist may not have been detected. Due to the smaller data set, results were not stratified according to age, sex, gender, ethnicity, education, or other substructure features of the participant population. Since data analysis was performed, the DSM IV-R version of DSM diagnostic assessment [110] has changed to the DSM V version. DSM V changed first-rank symptoms of auditory hallucinations and bizarre delusions to include positive symptoms of hallucinations, delusions, and disorganised speech. These symptoms were, however, captured by the Brief Psychiatric Rating Scale used in this study [111].

A further limitation is the absence of a training set; nevertheless, we have made a significant effort to describe all component biomarkers and demonstrate the sustained coherence of findings across several different forms of complementary data analysis. Since DSM IV diagnoses for schizophrenia and schizoaffective disorder are likely to co-aggregate with other mental illness diagnoses, biomarker comparison with other psychiatric diagnosis may have strengthened results. However, it is also possible that comparison across other diagnoses may hamper comparative strength, because symptomatic features associated with alternative diagnoses may overlap with schizophrenia and schizoaffective disorder conditions [119]. For this reason, we consider that a small exploratory case–control design with well-defined patients is preferable for the preliminary discovery of predictive biomarkers from candidate markers [118]. As discussed, S-adenosyl methionine (SAMe) is involved in the synthesis of creatine and creatinine; therefore, creatinine cannot be regarded as an independent biomarker in this project’s findings [120]. Despite excluding substance use and medications with potential to affect biomarkers, it was not possible to exclude participants who smoked cigarettes, whose biochemistry might be altered by inhaling nicotine. The identification of the HPLC elution Peak 1 is achieved via exclusion and identification from previously reported research on HPLC fractionation (Supplementary Materials, Section S4). Further research was impeded by the limitation of available standards. It is therefore unclear whether Peak 1-related correlates represent riboflavin degradation products or riboflavin-inhibiting analogues. Despite these limitations, the vitamin B2-related excretion findings in this study are significant, because vitamin B2 (riboflavin) is a precursor to FAD, which is required for *MTHFR* C677T activity and, therefore, for methylation [121]. 

It was not possible to exclude participants taking mood-stabilizing medications, and therefore, patients remained on these medications as needed. As noted in Section 5, one of these medications, sodium valproate, may raise homocysteine levels and lower folic acid levels [107]. Thyroid hormone levels were also not assayed in this study and have the potential to influence FMN synthesis from riboflavin [122]. Mental illness states are increasingly being recognised as whole-body metabolic diseases [123]; however, it is acknowledged that despite the genotype dependency of the biomarkers discovered in this study, the translation of results between urine and brain and body tissue are not yet fully understood. In this regard, it would be useful to measure biomarkers in both urine and blood compartments. It would also be useful to measure DNA expression levels and interactions of gene-coded enzymes in other pathways relating to neuroinflammation, glucose metabolism, hormone regulation, cell apoptosis, and glutamate neural transmission. The validation of this framework and its predictive biomarkers is required, and further research development will include different disease cohorts, medication-naïve prospective patients, blind ratings, and expanded assessment parameters.

## 8. Conclusions

The objective management of psychoses such as schizophrenia and schizoaffective disorder requires functionally meaningful and treatment-relevant biomarkers to confirm a diagnosis and aid clinicians in the diagnosis and treatment of patients with these conditions. Since the *MTHFR* C677T genotype has a known relationship with schizophrenia, this study presents predictive biomarkers for these conditions according to *MTHFR* C677T genotype. The findings of this study validate the original hypothesis, namely that a novel set of molecular and trace element markers related to *MTHFR* C677T genotypes, methylation pathways, and more remote pathways of methylation influence, can provide predictive biomarkers for the diagnosis of schizophrenia and schizoaffective psychosis and point towards therapeutic applications.

Relative amounts of excretion of riboflavin (vitamin B2) and its derivatives FMN and FAD, were found to play key roles in differentiating *MTHFR* C677T-related biochemical phenotypes. Riboflavin and its flavoprotein derivatives can affect activation of vitamin cofactors and influence the capability of *MTHFR* C677T genotypes to produce sufficient methyl folate to sustain methylation as well as influence enzyme activity in many proximal and remote biochemical pathways. These pathways influence dopamine and serotonin neurotransmitter availability and remotely modulate oxidative stress and inflammation responses. Single and group biomarkers discovered within these pathways relate to contrasting low- and high-methylation states that can predict diagnosis with greater than 90% sensitivity and specificity. These *MTHFR* C677T genotype-dependent biomarkers can advance diagnostic and prognostic certainty in the clinical management of psychosis. Their functional meaning opens a new field of methylation-related clinical education and psychosis subtyping, with potential for targeted approaches for the treatment of mental illness. After broader investigation and validation in larger cohorts, these findings have the potential to provide clinicians with functionally meaningful phenotypic descriptors and specific biomarker indices for monitoring illness progression, preventing relapse, informing treatment options, and overcoming treatment resistance in schizophrenia and schizoaffective psychosis. 

## Figures and Tables

**Table 1:** The *MTHFR* C677T genotype differentiated demographic data.

	*MTHFR* 677 Homozygous (TT Genotype) (5.2% Prevalence)	*MTHFR* 677 Heterozygous (CT Genotype) (48.5% Prevalence)	*MTHFR* 677 Negative (CC Genotype) (46.3% Prevalence)	*p* *
*n (*% of sample)	7 (100.0)	62 (100.0)	65 (100.0)	0.602
Age (males)	41.4 (35.6, 47.2)	43.4 (39.7, 47.2)	41.3 (37.9, 44.7)	0.692
Age (females)	33.0 (18.0, 62.7)	44.7 (40.0, 49.4)	44.0 (39.6, 44.7)	0.422
Age onset for cases	28.0 (17.7, 38.3)	22.1 (18.8, 25.5)	25.0 (20.6, 29.3)	0.636
Duration of illness (years) for cases	13.3 (1.2, 25.5)	18.4 (14.1, 22.7)	15.4 (11.4, 19.4)	0.346
BMI	29.1 (23.6, 34.5)	28.1 (26.1, 30.1)	28.2 (26.4, 29.9)	0.911
% Right-hand dominant	93.6 (85.3, 101.9)	91.0 (86.8, 95.1)	94.6 (91.1, 98.1)	0.539
Urine Creatinine	7.1 (3.9, 10.4)	9.7 (8.2, 11.2)	9.3 (7.8, 10.8)	0.618
Urine Vitamin B2	3.0 (0.2, 5.7)	10.4 (3.0, 17.8)	4.4 (2.5, 6.2)	0.175
Urine 5 Hyroxyindoleacetic acid (5-HIAA)	1.6 (1.0, 2.2)	2.3 (1.4, 3.1)	3.7 (2.2, 5.2)	0.537
Urine Dopamine (DA)	156.6 (111.8, 201.4)	137.3 (123.5, 151.1)	145.8 (132.9, 158.7)	0.521
Urine Noradrenaline (NA)	27.1 (19.7, 34.6)	25.3 (20.0, 30.6)	25.1 (20.9, 29.2)	0.356
Urine Adrenaline (AD)	3.4 (2.2, 4.6)	4.5 (3.1, 6.0)	4.4 (3.3, 5.5)	0.766
Serum Vitamin B6	315.0 (0.0, 729.8)	146.9 (114.9, 178.9)	115.0 (93.2, 136.9)	0.412
Plasma Red cell folate	1830.7 (1468.5, 2192.8)	1794.5 (1664.6, 1924.4)	1779.0 (1686.4, 1871.6)	0.847
Serum Vitamin B12	452.1 (357.3, 546.9)	421.6 (370.1, 473.2)	386.4 (348.6, 424.2)	0.393
Serum Vitamin D	59.0 (42.9, 75.1)	52.4 (47.4, 57.5)	51.8 (45.8, 57.8)	0.691
Serum Histamine	0.61 (0.41, 0.82)	0.69 (0.61, 0.76)	0.67 (0.58, 0.77)	0.795
SIR (symptom intensity rating)	82.3 (39.6, 125.0)	74.1 (64.5, 83.7)	73.2 (63.9, 82.6)	0.996
GAF (global function index)	71.4 (46.1, 96.8)	70.3 (63.4, 77.3)	67.8 (60.8, 74.7)	0.863

Mean value given with 95% confidence interval. * *p* based on the Kruskal–Wallace H test [16].

**Table 2:** *MTHFR* C677T genotype-dependent ROC results for vitamin B2/creatinine.

MTHFR C677T genotypes	*n*	Sensitivity %	Specificity %	PPV ^1^	NPV ^2^	OR ^3^	*p*	ROC ^4^ (AUC)	SE	*p*
**MTHFR 677 TT**										
Urine vitamin B2/creatinine level	6							1.000	0.000	0.000
Urine vitamin B2/creatinine ROC	6	100	100	100	100			1.000	0.000	0.000
**MTHFR 677 CT**										
Urine vitamin B2/creatinine level	55					0.90	0.433	0.533	0.081	0.341
Urine vitamin B2/creatinine ROC	55	56.0	66.7	0.8	99.7	2.55	0.095	0.613	0.067	0.045
**MTHFR 677 CC**										
Urine vitamin B2/creatinine level	61					1.08	0.796	0.521	0.075	0.388
Urine B2/creatinine ROC	61	32.3	70.0	0.5	99.6	1.11	0.849	0.511	0.060	0.426

^1^ PPV = positive predictive value, ^2^ NPV = negative predictive value, ^3^ OR = odds ratio, for population prevalence of schizophrenia of 0.45% (0.0045). ^4^ ROC = receiver operating characteristics, where area under the (ROC) curve (AUC) is >0.9, and candidate marker diagnostic discrimination between cases and controls is excellent. AUC 0.7–0.8 = acceptable discrimination. AUC 0.5 to 0.7 = poor diagnostic discrimination.

**Table 3:** *MTHFR* 677 TT genotype-dependent ROC biomarkers.

*MTHFR* 677 TT-Related Biomarkers (*n* = 7)	AUC	AUC *p*	Explicit Biochemical Linkage with High and Low Methylation States
Urine excretion vitamin B2 ug/L	1.000	0.0000	FAD conservation by dissociation from the inactive MTHFR enzyme conserves FMN and riboflavin precursors, as described in Section 2.2.1.
Urine vitamin B2/creatinine	1.000	0.0000	Riboflavin, expressed in relation to creatinine has 100% predictive sensitivity and 100% predictive specificity. Since creatinine is a measure of renal excretion, this points to the renal conservation of riboflavin as an important explanatory mechanism for riboflavin’s ability to predict diagnosis in the *MTHFR* 677 TT genotype, as described in Section 2.2.1.
Peak 2 amplitude/Peak 1 amplitude	1.0000	0.0000	Riboflavin (Peak 2) is more than its unidentified co-analyte band (Peak 1), which is presumed to be riboflavin-related metabolites (Section 2.2.1).
Peak 2 area/Peak 1 area	1.0000	0.0000	As above.
Elevated % free Copper (Cu) to Zinc (Zn) ratio	0.916	0.0000	Zn and Cu have a reciprocal relationship in plasma and tissues [37]. There is potential for Zn to be over-utilised by the compensatively activated BHMT pathway required to offset the low methylation conditions brought about by the inactivity of the MTHFR thermolabile enzyme.
Elevated levels of serum B12	0.750	0.0416	MTHFR enzyme inactivity deprives MS of its 5-MTHF co-factor, leaving its B12 cofactor unutilised. Also, unutilised FMN reactivates vitamin B12.
Elevated [vit B12 × % free Cu × Homocysteine/[Zinc × folate × vit B6]	1.000	0.000	Elevated Cu impedes CBS activity and there is insufficient 5-MTHF for MS activation; therefore, homocysteine is elevated. Reason for elevated vitamin B12 is as discussed in line above. Zinc is low because is overutilized as a cofactor by BHMT, Low folate is due restricted 5-MTHF product due to the thermolabile, MTHFR 677 TT genotype coded MTHFR enzyme. Low vitamin B6 may be due to its over-utilisation by serine hydroxy methyltransferase (SHMT) and other enzymes in serine metabolic pathways. The serine metabolism of these pathways is enhanced because the inactive MTHFR enzyme pathway for serine metabolism is restricted in this genotype.
Elevated [vitamin B12 × % free Cu × Homocysteine]/[Zinc × folate × vit B6 × vitamin D]	1.000	0.000	Elevated Cu, vitamin B12, and low vitamin B6 and Zn explained above. Vitamin B12/Vitamin D proved to be a distinctive biomarker for low methylation, as discussed in Section 2.3.4.

Biomarkers representing effects of the underlying low methylation state attributed to the MTHFR 677 TT genotype coding for an MTHFR enzyme with restricted activity are presented in blue.Biomarkers attributed to compensative high methylation state are presented in red. / = divided values (ratios). × = multiplied values.

**Table 4:** *MTHFR* 677 CC-dependent ROC biomarkers.

*MTHFR* 677 CC-Dependent ROC Biomarkers	AUC	AUC *p*	Explanation in Terms of Low Methylation Dynamics
AD/MHMA	0.8596	0.0000	Monoamine oxidase inhibition from lack of FAD cofactor.
NA/MHMA	0.7742	0.0000	Monoamine oxidase inhibition from lack of FAD cofactor.
High histamine	0.6103	0.0078	Activated vitamin B6 is required for histamine metabolism.
Elevated urine 5-HIAA levels	0.7211	0.0000	In a low riboflavin setting, FAD synthesis is restricted and FMN is similarly unavailable for vitamin B6 activation to pyridoxine phosphate (PLP). There is insufficient FAD and B6 (PLP) for activity of the tryptophan pyrrolase enzyme at the head of the kynurenine pathway. Consequently, the alternative metabolic pathway for L tryptophan is preferred and L tryptophan is converted to serotonin, the metabolite of which can overflow into the urine as 5-HIAA (Scheme 1).
DA × 5-HIAA	0.7386	0.0000	5-HIAA is elevated for the above reasons. Dopamine is elevated because the lack of FAD restricts DA metabolism by MAO-B, and SAMe unavailability restricts NA conversion to AD downstream of DA.
Low red cell folate levels	0.6563	0.0036	Excess riboflavin inhibition by metabolites means that its FAD product is relatively unavailable for the activation of folate to 5-MTHF.
Low vitamin levels of vitamin B6	0.6951	0.0001	FMN availability from FAD is also limited by riboflavin inhibition by metabolites and FMN is required for vitamin B6 activation.
Low vitamin D levels	0.6719	0.0012	Vitamin D requires FAD for activation.
Vitamin B12/vitamin D	0.6719	0.007	Where there is insufficient Vitamin B2 precursor for the synthesis of FAD and insufficient cofactor for the *MTHFR* C677T gene, 5-MTHF is lacking for methionine synthase activation and its related cofactor vitamin B12 is unutilised and accumulates. Vitamin D activation is restricted in a low FAD setting.
[Vitamin B12 × Homocysteine × Cu%/Zinc × folate × vitamin B6 × vitamin D]	0.6462	0.0314	Explanation in Section 2.3.1 below.
High % free copper to zinc ratio	0.6406	0.0017	Elevated free copper released by DBH enzyme (see Section 2.3.1, below), in a setting of reciprocal relationship between copper and zinc [37].

Biomarkers considered to be associated with low methylation and low vitamin B2 and B6 phenotypes are presented in blue.

**Table 5:** Logistic regression for *MTHFR* 677 CC-dependent biochemical variables.

Logistic Regression for MTHFR 677 CC			Observations 57 Likelihood Ratio Chi-square (4) 45.7		Prob > Chi-square p-value 0.000 Pseudo-R2 0.579	
Case Certainty CC *n*57	Odds Ratio (OR)	Std. Err.	z *	P > |z|	[95% Conf. Interval]	
Vitamin B6	0.9747	0.0122	−2.03	0.042	0.9511	0.9990
Peak 1/Peak 2	0.1738	0.1643	1.85	0.064	0.0272	1.1082
AD + NA/MHMA	1.2504	0.0909	3.07	0.002	1.0843	1.4419
5-HIAA	1.8168	0.6930	1.57	0.118	0.8602	3.8371

* z is coefficient/standard error (Std. Err.), where the OR (odds ratio) is an exponential of the coefficient of predictor variables. P > [z] = probability associated z factor at 95% confidence level. Peak 1 amplitude = riboflavin co-analyte (presumed degradation product); Peak 2 amplitude = riboflavin. Biomarkers considered to be representative of an under-methylation process are presented in blue.

**Table 6:** Significant ROC and odds ratio characteristics for variables within the *MTHFR* 677 CT genotype.

Variable for MTHFR 677 CT	*n*	Sensitivity %	Specificity %	PPV %	NPV %	OR	OR *p* Value	AUC	SE *	*p*
5HIAA	61							0.6086	0.0683	0.0559
High 5HIAAROC	61	30.0	93.5	2.1	99.7	6.2	0.028	0.6177	0.0481	0.0072
NA/DA	61							0.7876	0.0580	0.0000
High NA/DA ROC	61	80.0	67.7	1.1	99.9	8.4	0.000	0.7387	0.0566	0.0000
NA/MHMA	60							0.8144	0.0567	0.0000
High NA/MHMA ROC	60	76.7	80.0	1.7	99.9	13.1	0.000	0.7833	0.0541	0.0000
High Histamine ROC	62	45.2	77.4	0.9	99.7	2.8	0.064	0.6129	0.0593	0.0285
Vit D	61							0.6269	0.0723	0.0396
Low Vit D ROC	61	70.0	58.1	0.7	99.8	3.2	0.030	0.6403	0.0620	0.0118
Red cell folate	62							0.6774	0.0686	0.0049
Low (red cell) Folate ROC	62	67.7	64.5	0.9	99.8	3.8	0.013	0.6613	0.0611	0.0041
Serum B12	62							0.6098	0.0727	0.0655
High B12 ROC	62	54.8	67.7	0.8	99.7	2.6	0.076	0.6129	0.0623	0.0350
Low B6 ROC	59	76.7	44.8	0.6	99.8	2.7	0.085	0.6075	0.0612	0.0395

Biomarkers considered to be representative of a low methylation process are presented in blue. *p* = significance at 95% confidence. The critical *p* value for AUC for the CT genotype was 0.0304 and the critical *p* value for OR estimates for the CT genotype was 0.019. *SE = Standard Error.

**Table 7:** Summary of correlative findings for biomarkers across all *MTHFR* C677T genotypes, according to low or high methylation.

Genotype	*MTHFR* 677 TT	*MTHFR* 677 CT	*MTHFR* 677 CC
	Primarily Low Methylation, Compensated by High Methylation	Low and Mixed Methylation	Low Methylation
Case Prevalence	5.2%	46.3%	48.5%
Vitamin B2 (riboflavin) *	Elevated vitamin B2 ** Vitamin B2/creatinine *	B2/creatinine ROC *	none
Plasma homocysteine	High plasma homocysteine ROC	High plasma homocysteine ROC	
5-HIAA (serotonin Metabolite)	Low 5-HIAA	High 5-HIAA excretion level	High 5-HIAA excretion level
Histamine		High histamine ROC	High histamine
Vitamin B2	Elevated serum B12	Mixed features of TT and CC genotypes	Low RC Folate: *n*64*, rho* −0.245*, p* 0.051 Low vitamin B6: *n*63*, rho* −0.335*, p* 0.007 Low vitamin D ROC*:* *n*64*, rho* −0.344*, p* 0.005
High catecholamines	Elevated AD/NA ratio	Mixed features of TT and CC genotypes	Restricted catecholamine metabolism AD/MHMA, NA/MHMA, DA X 5HIAA
**Best compound biomarker correlates for** **differentiating functional methylation states.**	**Elevated DA ROC** **Elevated vitamin B12** **Elevated urine B2** **Vitamin B2/creatinine** **Elevated AD/NA ratio** **Low zinc**	**DA X 5HIAA ROC** **NA + AD/ MHMA** **vitamin B12/vitamin D ROC ** **5-HIAA**	**NA/AD** **NA/MHMA and AD/MHMA** **High histamine** **Vitamin B12/vitamin D** **5-HIAA**

* derived from urinary excretion, where Peak 2 = vitamin B2 (riboflavin) and Peak 1 = vitamin B2 metabolite. ** derived from Peak 2/Peak 1 ratio for amplitude and area under HPLC elution curve. Biomarkers considered to be representative of an under-methylation process are presented in blue. Biomarkers attributed to compensative high methylation state are presented in red.

**Table 8:** Summary of all biomarker findings across the three *MTHFR* C677T genotypes with functional explanations and symptom affiliations.

	Homozygous *MTHFR* 677 TT (5.2%)	Heterozygous *MTHFR* 677 CT (46.3%)	Polymorphism Free *MTHFR* 677 CC (48.5%)
**Methylation State**	Low methylation with High compensative methylation	Mixed	Low methylation state
**Functional Explanation**	MTHFR enzyme disability initially restricts provision of 5-MTHF, which restricts SAMe synthesis (methylation) by usual route (under methylation). This state necessitates high compensative methylation driven by BHMT enzyme. Also, flavoproteins (FMN, FAD) dissociate away from the disabled MTHF enzyme. Together, high SAMe and FAD facilitate catecholamine metabolism and deplete neurotransmitters. FAD also facilitates NAD synthesis. FAD and FMN facilitate activation of other vitamin cofactors necessary for oxidative defence and indole-catecholamine metabolism.	Mixed or alternating functional processes	High riboflavin degradation products and low riboflavin with low flavoproteins (FMN FAD). Low riboflavin restricts MTHFR enzyme activity through restricted provision of flavoprotein (FAD cofactor), and thereby restricts folate production, leading to low methylation state. Together low riboflavin, reduced FAD and low SAMe, restrict indole catecholamine metabolism raising neurotransmitter levels. Low FAD and FMN inhibit vitamin activation. Low vitamin B6 activation inhibits glutathione synthesis, and low FAD inhibits glutathione restoration. Low vitamin B2 related to gastrointestinal inflammation sets in motion a vicious cycle of low riboflavin and low vitamin activation.
**Biomarker Outcomes**	Vitamin B2 sufficiency. Adequate activated vitamins. High DA. AD/NA. Low zinc.	Mixed biomarkers	Low vitamin B2. Low activated vitamins. High catecholamines. NA/AD. High histamine. High Serotonin secretion.
**Biomarker Symptom relationships**	Generally higher function with fewer symptoms. Manic symptoms and sudden depression onset due to catecholamine depletion. (May relate to bipolar affective mood state)	May relate to mixed or alternating mood states or rapid cycling bipolar state	Conserved neurotransmitters promote high arousal states, but concurrent low activated vitamin, oxidative stress, and low methylation stall biochemistry processes. May relate to chronic fatigue and negative symptoms in schizophrenia.

## Data Availability

Limited anonymous data are available from the corresponding author upon reasonable request.

## Supplementary Materials

The supporting information can be downloaded at: https://www.mdpi.com/article/10.3390/ijms242115845/s1.

## Abbreviations

B2	Vitamin B2 (riboflavin)
B6	Vitamin B6 (Pyridoxal Phosphate (PLP) is active form)
*MTHFR* C677T gene	gene, coding for 5,10-methylenetetrahydrofolate reductase enzyme
MTHFR enzyme	5,10—methylenetetrahydrofolate reductase enzyme.
5-MTHF	5—methyltetrahydrofolic acid (active form of folate)
5-HIAA	5-hydroxyindoleacetic acid
AD	Adrenaline
DA	Dopamine
NA	Noradrenaline
BHMT	Betaine-homocysteine S-methyltransferase
BPRS	Brief Psychiatric Rating Scale
Case-ness	DSM—diagnostic identification for schizophrenia and schizoaffective disorder
CBS	Cystathionine beta synthase.
COMT	Catechol-O-methyltransferase
DAO	Diamine oxide
DBH	Dopamine beta-hydroxylase
DMG	Dimethylglycine, TMG Trimethylglycine
DNMT1	DNA methyltransferase 1
FAD	Flavin dinucleotide
FMN	Flavin mononucleotide
GSH	Reduced (active) glutathione
GSSH	Oxidised form of glutathione
HCY	Homocysteine
HO	Haeme oxidase
HPL	Hydroxyhaemopyrroline-2-one
HPLC	High-pressure liquid chromatography
KP	Kynurenine acid pathway; KYNA—Kynurenic acid
MAO	Monoamine oxidase
MHMA	Methylhydroxymandelic acid
MS	Methionine Synthase
OR	Odds Ratio
PANSS	Positive and Negative Syndrome Scale
Peak 1	first HPLC elution peak in urine riboflavin analysis = unidentified riboflavin co-analyte (presumed metabolite)
Peak 2	second peak in riboflavin HPLC urine analysis = riboflavin (standard)
PLP	pyridoxal-5-phosphate (activated form of vitamin B6)
PPV	positive predictive value; NPV—negative predictive value
SAH	S-adenosyl-homocysteine
SAHH	S-adenosyl homocysteine hydrolase enzyme
SAMe	S-adenosylmethionine
SHMT	Serine hydroxymethyl transferase
SOD	Superoxide dismutase.
TP	Tryptophan pyrrolase
TSP	Transsulfuration pathway
B6	Vitamin B6 (activated form pyridoxal-5-phosphate PLP)
B12	Vitamin B12 (Cobalamin)
Vitamin D	activated 25-OH form of vitamin D
