# Supplementary material for: Molecular Mechanism Biomarkers Predict Diagnosis in Schizophrenia and Schizoaffective Psychosis, with Implications for Treatment
Source: Int J Mol Sci. 2023 Oct 31;24(21):15845. doi: 10.3390/ijms242115845 (PMC10650772; doi:10.3390/ijms242115845)
Supplement: Supplementary file 1 [file ijms-24-15845-s001.zip › ijms-2617470-supplementary.pdf]

# Supplementary

*Original Research.*

# Molecular mechanism biomarkers predict diagnosis in schizophrenia and schizoaffective psychosis, with implications for treatment

**Stephanie Fryar-Williams [1,2,3,4,*], Graeme Tucker [5], Jörg Strobel [6], Yichao Huang [7] and Peter Clements [7,8,*]**

1 Youth in Mind Research Institute, Unley, SA 5061, Australia
2 The Queen Elizabeth Hospital, Woodville, SA 5011, Australia
3 Basil Hetzel Institute for Translational Health Research, Woodville, SA 5011, Australia
4 Department of Nanoscale BioPhotonics, Faculty of Health and Medical Sciences, School of Biomedicine, The University of Adelaide, Adelaide, SA 5000, Australia
5 Department of Public Health, Faculty of Health and Medical Sciences, Adelaide Medical School, The University of Adelaide, Adelaide, SA 5000, Australia
6 Department of Psychiatry, Faculty of Health and Medical Sciences, Adelaide Medical School. The University of Adelaide, Adelaide, SA 5000, Australia
7 Waite Research Institute, The University of Adelaide, Urrbrae, SA 5064, Australia
8 Department of Paediatrics, Faculty of Health and Medical Sciences, Adelaide Medical School, The University of Adelaide, Adelaide, SA 5000, Australia
* Correspondence: stephanie.fryar-williams@adelaide.edu.au; peter.clements@adelaide.edu.au

**Contents**

**S 1. Inclusion criteria........ 2**
**S 2. Exclusion criteria........ 2**
**S 3. Rating measures........ 3**
**S 4. Laboratory Assays including details of urine riboflavin analysis. ........ 4**
**S 5. Data characteristics for MTHFR C677T data sets. ........ 8**
**S 6. Significant Spearman's correlate relationships within the MTHFR 677 TT genotype. ........ 10**
**S 7. Significant Spearman's correlate relationships within the MTHFR 677 CC genotype. ........ 11**
**S 8. Cross tab analysis for MTHFR 677 TT dependent variables........ 13**
**S 9. Logistic regression analysis for MTHFR 677 CC dependent biochemical variables........ 14**
**S 10. Significant case-correlates within the MTHFR 677 CT genotype........ 15**

**S 1.	Inclusion criteria.**

| Inclusion Criteria | Method |
|---|---|
| Orientated in time, place and person. Non-detained under Mental Health Act Capacity to consent | Clinical examination and case-note examination |
| Diagnosis of schizophrenia or schizoaffective disorder, made by a consultant psychiatrist in the ward or community satellite clinic setting, according to the DSM-IV-R classification criteria and checked against DSM-IV-R symptom checklist at the recruitment stage | American Psychiatric Association: Diagnostic and Statistical Manual of Mental Disorders. (4th Ed. Rev) (DSM IV-R). American Psychiatric Association 1994. Washington DC. 17. Simpson GM, Angus JWS. A rating scale for extrapyramidal side effects. Acta Psychiatry Scand. 1970; 212 (44):11–9. |
| Absence of rigidity, dyskinesia, tremor or instability in ocular-muscle dysfunction, hand, forearm and shoulder. | Clinical examination: Simpson GM, Angus JWS. (1970) A rating scale for extrapyramidal side effects. *Acta Psychiatrica Scandinavica*, **212** (44), 11-19. |
| Zuclopenthixol, Modecate, Amisulpride Risperidone, Quetiapine, sodium valproate. | Treatment chart and case-note examination |
| Risk factors of interest: such as family history of mental illness, history of emotional or physical abuse, developmental disorder, learning disorder or subclinical head injury. | Case-note examination and personal inquiry. |

**S 2.	Exclusion criteria**

| Exclusion Criteria | Method |
|---|---|
| Lack of orientation in time, place and person.<br>Detained under Mental Health Act<br>Lacking consent capacity | Clinical examination and case-note examination |
| Alternative diagnosis | American Psychiatric Association: Diagnostic and Statistical Manual of Mental Disorders. (4th Ed. Rev) (DSM IV-R). American Psychiatric Association 1994. Washington DC. 17. Simpson GM, Angus JWS. A rating scale for extrapyramidal side effects. Acta Psychiatry Scand. 1970; 212 (44):11–9. |
| Unstable pharmacotherapy over assessment period. | Treatment chart and case-note examination |
| Extra-pyramidal side effects in ocular, hand, forearm, and shoulder | Clinical examination; Simpson GM, Angus JWS. (1970) A rating scale for extrapyramidal side effects. Acta Psychiatrica Scandinavica, 212 (44), 11-19. |

| | |
|---|---|
| Clozapine, Olanzapine, antihistamines, or vitamin therapy | Treatment chart, case-note examination, and personal questioning. |
| Recent or unresolved history or positive test for alcohol or other substance abuse. | Treatment chart, case-note examination, and personal questioning |
| Upper respiratory tract infections | Treatment chart, case-note examination, and personal questioning |
| Intellectual disability or clinically documented or descriptive history of head injury with unconsciousness or hospitalisation. | Treatment chart, case-note examination, and personal questioning. |

**S 3. Rating measures**

| Functional Rating Scale | Citation |
|---|---|
| The Brief Psychiatric Rating Scale (BPRS) | Overall JE and Gorham DR (1962) The Brief Psychiatric Rating Scale. Psychological Report 10(3): 799-812. |
| Positive and Negative Syndrome Scale for schizophrenia (PANSS) | Kay SR, Fiszbein A and Opler LA (1987) The positive and negative syndrome scale (PANSS) for schizophrenia. Schizophrenia Bulletin 13(2): 261-276. |
| Clinical Global Impression of Severity (CGI) | Guy W (1976) Clinical global impressions [CGI]. ECDEU assessment manual for psychopharmacology. National Institute of Mental Health, Early Clinical Drug Evaluation, Psychopharmacology Research Branch, Rev. Rockville, MD, U.S. |
| Global assessment of Function (GAF) | Frances A, Incus HA, and First MB (1994) Diagnostic and Statistical Manual of Mental Disorders, 4th edition, American Psychiatric Association, Washington, DC. |
| Social and Occupational Functioning Assessment Scale (SOFAS) | Goldman HH, Skodol AE and Lave TR (1992) Revising axis V for DSM-IV: a review of measures of social functioning. American Journal Psychiatry 149(9): 1148-1156. |

**S 4. Laboratory Assays including details of urine riboflavin analysis.**

| Neuro-Biochemistry | All fasting blood samples were collected between 9 and 11 am daily.<br>By arrangement, transported directly to the laboratory with no storage. | Laboratory/Reference |
|---|---|---|
| Serum Vitamin D (25-OH) | Diasorin Liason assay kit, for use on the Liaison platform. (nmol/L) | Clinpath Laboratories,<br>19 Fullarton Rd,<br>Kent Town.<br>South Australia 5067<br>+61 8 8366 2000. |
| Serum total Vitamin B12 | Competitive Electrochemiluminescent Immunoassay. Roche Modular E 170 Automated Immunoassay Analyser, using Roche Vitamin B12 Reagent. (nmol/L) | Clinpath Laboratories.<br>As above. |
| Plasma Red Cell Folate | Competitive Electrochemiluminescent Protein Binding Assay, using Roche Modular E 170, using Roche Elecsys Folate III assay on Automated Immunoassay Analyser. (nmol/L) Pfeiffer *et al.* (2004) and Roche indications are that for majority of patient samples with Total Folate of < 50 nmol/L, consisted of average, 93.3% 5-Methyl-THF, 2.3% Folic Acid, and 4.4% 5-Formyl-THF while samples with Total Folate of >50 nmol/L had, on average, 81.7% 5MeTHF, 15.7% FA, and 2.5% 5FoTHF. | Clinpath Laboratories. |
| Serum Vitamin B6 (Pyridoxal-5′-phosphate coenzyme form) | Whole blood High Pressure Liquid Chromatography with fluorescent detection. Chromsystems Vitamin B6 in Whole Blood High pressure Liquid Chromatography Reagent Kit. Waters Alliance 2695 Separations Module. Waters 474 Fluorescence Detector(nmol/L) | Sullivan Nicolaides Pathology<br>143 Whitmore St,<br>Taringa.<br>Queensland 4068. Australia.<br>+61 7 337 8666 |
| Serum Copper | Flame Atomic Absorption Spectrophotometry. Varian AA-240FS. (umol/L) | Douglass Hanly Moir Pathology<br>14 Griffnock Avenue,<br>Macquarie Park.<br>New South Wales 2113. +61 2 98555222 |
| Plasma Red Cell Zinc | Inductively coupled plasma mass spectroscopy (ICP-MS), using 6% n-Butanol reagent and Agileny ICP-MS 7500ce analyser. (umol/L) | Sullivan Nicolaides Pathology. |

| | | |
|---|---|---|
| Serum Ceruloplasmin | Immunoturbidimetric method, using 6K91-30 Multignet Caeruloplamin Kit and Abbott Architect ci16000 analyser. (g/L) | Douglass Hanly Moir Pathology. |
| Percentage Free Copper/Red Cell Zinc | Percentage of free copper in the serum calculated by an equation based on the molecular and atomic weights of ceruloplasmin and copper (one ceruloplasmin molecule binds to six copper atoms). The ratio of the percentage free copper to red cell zinc was calculated as “percentage free copper” / “Red cell zinc umol/L”. | Calculated by authors |
| **Intermediate substrates and enzymes** | | |
| MTHFR Ala222Val (C677T) methyl tetrahydrofolate reductase polymorphism | Peripheral blood samples were collected to assay for MTHFR Ala222Val (C677T) methyl tetrahydrofolate reductase polymorphism, from which red cells were lysed and DNA was isolated from residual lymphocyte fraction.<br>Real time PCR analysis method.<br>Roche Diagnostics Light-Cycler 480 kit. Using TecnoBiol reagents, Sigma probes and primers on Roche LC480 analyser | Douglass Hanly Moir Pathology. |
| Plasma homocysteine | Ice transported EDTA sample. Competitive Chemiluminescent Immunoassay, using Siemens Homocysteine reagent on Siemens Advia centaur Automated Immunoassay (umol/L). | SA Pathology.<br>+61 8 8222 3000 |
| Serum histamine | Beckman Coulter Radio Immunoassay, using Beckman Coulter R.I.A. Kit on Perkin Elmer Wizard 1470 Automated Gamma Counter. (umol/L) | Sullivan and Nicolaides. |

| **Urine Tests Neurotransmitters** | **Method, Analyzer, Reagents.** | **Laboratory/Reference** |
|---|---|---|
| Biogenic amines: Dopamine, Noradrenaline and Adrenaline, | Spot-baseline (fasting) urinary neurotransmitter testing (second void morning), snap-frozen to minus 30 degrees and analysed by mass spectrometry, using nanomoles per millimole of urinary creatinine as a standard. | SA Pathology, Adelaide, South Australia.<br><br>Whiting MJ. 2009. Simultaneous measurement of urine metanephrines and catecholamines by liquid chromatography with tandem mass spectrometric detection. *Annals of Clinical Biochemistry*, **46**:129-136 |
| Creatinine | Spot urine specimen from the same void as biogenic amines, expressed in (millimoles per Litre) | SA Pathology, Adelaide SA. |
| Oxidative stress: Urinary hydroxyhemopyrroline-2- one | Fasting urine sample collected whilst patient at rest, separated from blood drawing by minimum of 2 hours. | Applied Analytical laboratories, Meadowbrook, Queensland. |
| ((Urine Riboflavin ug/L | Ultra performance liquid chromatography (HPLC), with fluorometric detection of the eluted riboflavin Peak (1) riboflavin metabolites and peak (2) = riboflavin. | Lipid analysis laboratory<br>Waite Agricultural Research Institute<br>Adelaide SA. |

**Urine analysis Method for riboflavin and its metabolites**

Phosphorylation of riboflavin maintains its metabolic trapping in tissues where it is mostly enzyme bound as flavoproteins like FMN and FAD. Gastaldim G.; Ferrari, G.; Verri, A.; Casirola, D.; Orsenigo, M.N.; Laforenza, U. Riboflavin phosphorylation is the crucial event in riboflavin transport by isolated rate enterocytes. *J Nutr* **2000**,*130*,2556–61. Unbound flavins are rapidly hydrolysed to free riboflavin, which diffuses from cells and is excreted in the urine as riboflavin or other metabolites, such as 7-hydroxymethylriboflavin (7-$\alpha$-hydroxy riboflavin). There is a linear relationship between urinary recovery of riboflavin and its metabolites and riboflavin absorption (bioavailability) and urinary riboflavin recovery can serve as an index of riboflavin absorption (Morrison, A.B.; Campbell, J.A. Vitamin absorption studies. I Factors influencing the excretion of oral test doses of thiamine and riboflavin by human subjects. *J Nutr*. **1969**, *72*, 435-440. West. D/W.; Owen, E.C. The urinary excretion of metabolites of riboflavine by man. *Brit. J. Nutrition*. **1960**, *23*,889-898). Some urinary metabolites reflect bacterial activity in the gastrointestinal tract (Singer, T.P.; Kenney, W.C.; Biochemistry of covalently bound flavins. *Vitam Horm* **1974**,*32*,1–45. Chastain, J.L.; McCormick, D.B. Flavin catabolites: identification and quantitation in human urine. *Am J Clin Nutr* **1987**,46,830–4).

**Method: Ultra performance liquid chromatography (HPLC) with fluorometric detection of the eluted riboflavin peak (1, 2)**

Standardization procedure: Riboflavin standard was from Sigma Aldrich. Stock riboflavin standard solution (1 mg/mL) was prepared in 10% acetonitrile and stored in -80 °C freezer. The stock solution was diluted periodically as needed from which a new standard curve freshly made for each batch. Working solution with range between range between 4-20 mg/L, was prepared monthly from the stock and store in fridge. Standard curve was obtained by independently diluted levels of riboflavin from one stock solution, and the resulting peak area were then plotted against riboflavin concentrations.

Sample procurement and preparation procedure: 15-50 ml urine was collected in the early morning after overnight fast and immediately frozen at -20 degrees C and stored at this temperature with protection from light. 170 μL thawed urine was added to 30 μL of acetonitrile in a 1.5 mL Eppendorf tube and incubated at 4 °C for 30 mins. The tube was then centrifuged at 13000 rpm, 10 °C for 10 mins. The supernatant was transferred into a 96-well plate (Greiner, Australia), and 5 μL were injected on to HPLC to be analysed.

Details of HPLC procedure: The HPLC used Agilent 1260 Infinity system equipped with Agilent ZORBAX Eclipse Plus C18 column (RRHD 3.0×50mm, 1.8 μm) with guard column with a flow rate of 0.5 mL/min. The chromatographic separation was performed at 25 °C and used gradient elution with mobile phase A consisting of 1% acetonitrile and B of 50% acetonitrile. The gradient started with 0% B and ramped up to 100% at 2 mins and maintained for 0.9 mins. The mobile phase was then equilibrated to initial condition (at 3 min) before the next injection. Riboflavin was measured with a fluorescence detector ($\lambda_{Ex}$: 445nm and $\lambda_{Em}$: 530 nm). Samples with a very high riboflavin level (N=2) required extra dilution. Agilent OpenLAB was used for instrument control and data analysis. \

Outcome: Two ranges of fractions were obtained which were separated at 2 mins. According to literature, the first range (at 0.4 mins to 1.5 mins) is riboflavin metabolites, with the main preceding peak being reported in literature as 7-7$\alpha$-hydroxy riboflavin (Shodex HPLC Column homepage https://www.shodex.com/en/dc/05/02/06.html#!) After this peak, riboflavin was detected at 3.35 mins with FMN and FAD eluting at its leading edge. Identification of HPLC elution Peak 1, is by exclusion and from previously reported research identifying the molecule as 7-7$\alpha$-hydroxy riboflavin on HPLC fractionation. Further identification was impeded by limitation of available standards. It is therefore unclear whether Peak 1 co-analyte represents reduced riboflavin through analog inhibition or degradation.

S 5. Data characteristics for MTHFR C677T data sets (IBM Corp. Released 2021. IBM SPSS Statistics for Windows, Version 28.0. Armonk, NY: IBM Corp)

| | **MTHFR TT** | **homozygous** | | **MTHFR CT** | **heterozygous** | | **MTHFR CC** | | |
|---|---|---|---|---|---|---|---|---|---|
| | Mean | N | Std. Deviation | Mean | N | Std. Deviation | Mean | N | Std. Deviation |
| 5 HIAA | 1.57 | 7 | 0.787 | 2.26 | 61 | 3.371 | 3.71 | 65 | 6.269 |
| NA/DA | 0.18 | 7 | 0.064 | 0.18 | 61 | 0.112 | 0.18 | 65 | 0.155 |
| AD/NA | 0.13 | 7 | 0.048 | 0.18 | 61 | 0.154 | 0.17 | 65 | 0.120 |
| NA/MHMA | 1978.57 | 7 | 1237.557 | 1481.11 | 60 | 751.035 | 1646.30 | 63 | 1017.576 |
| % Free Cu/Zn | 0.14 | 7 | 0.440 | 0.29 | 62 | 0.465 | 0.26 | 64 | 0.585 |
| AD/MHMA | 2.50 | 7 | 1.893 | 2.70 | 60 | 2.796 | 2.89 | 63 | 2.990 |
| Vitamin B12 /vit. D | 8.99 | 7 | 4.953 | 9.77 | 61 | 7.796 | 9.17 | 64 | 5.778 |
| Vitamin B12/ folate | 0.26 | 7 | 0.086 | 0.25 | 62 | 0.147 | 0.23 | 64 | .101 |
| Vitamin B12 / zinc | 33.09 | 7 | 10.160 | 31.11 | 62 | 14.153 | 28.95 | 65 | 12.429 |
| Vitamin B12 / zinc X folate | 0.02 | 7 | 0.007 | 0.02 | 62 | 0.010 | 0.02 | 64 | 0.008 |
| Vitamin B12 x Cu / Zn X folate | 0.03 | 7 | 0.105 | 0.06 | 62 | 0.112 | 0.05 | 64 | 0.173 |
| Vitamin B12 x Cu / zinc x folate x vit B6 | 0.00 | 7 | 0.001 | 0.00 | 59 | 0.001 | 0.00 | 62 | 0.004 |
| Vitamin B12 x Cu X homocysteine / zinc x folate x vit B6 | 0.00 | 7 | 0.000 | 0.00 | 57 | 0.000 | 0.00 | 61 | 0.002 |
| Plasma Homocysteine | 10.87 | 7 | 4.816 | 9.64 | 61 | 1.869 | 9.70 | 64 | 2.426 |
| Histamine | 0.61 | 7 | 0.273 | 0.69 | 62 | 0.304 | 0.67 | 65 | 0.381 |
| Vitamin D | 59.00 | 7 | 21.510 | 52.43 | 61 | 20.015 | 51.78 | 64 | 24.280 |
| Red Cell folate | 1830.71 | 7 | 484.247 | 1794.52 | 62 | 517.123 | 1779.00 | 64 | 374.541 |
| Serum B12 | 452.14 | 7 | 126.796 | 421.65 | 62 | 205.263 | 386.43 | 65 | 154.087 |
| Vitamin B6 | 315.00 | 7 | 554.519 | 146.86 | 59 | 124.183 | 115.04 | 63 | 87.609 |
| HPL/Creatinine | 2.27 | 7 | 1.590 | 4.37 | 61 | 5.601 | 5.04 | 65 | 6.517 |
| HPL/SG | 15.91 | 7 | 12.948 | 34.15 | 61 | 41.714 | 45.50 | 65 | 79.182 |

| | | | | | | | | |
|---|---|---|---|---|---|---|---|---|
| Urine creatinine | 7.11 | 7 | 4.346 | 9.67 | 61 | 5.872 | 9.29 | 65 | 5.944 |
| Vitamin B2 | 2.9748 | 6 | 3.37918 | 10.3920 | 55 | 27.67252 | 4.3641 | 61 | 7.43990 |
| Peak Region 1 amplitude | 0.5677 | 6 | 0.29610 | 0.9870 | 54 | 1.14692 | 0.8631 | 61 | 0.64584 |
| Peak Region 1 area under peak | 6.3711 | 6 | 3.09404 | 11.3988 | 54 | 12.45771 | 9.0003 | 61 | 5.27581 |
| Peak Region 2 amplitude | 4.8302 | 6 | 3.00026 | 14.0674 | 54 | 20.87696 | 8.4740 | 61 | 10.46451 |
| Peak Region 2 area under peak | 19.0015 | 6 | 9.33328 | 51.7247 | 54 | 70.62112 | 30.8188 | 61 | 31.23222 |
| Dilution Corrected B2 | 5.6226 | 6 | 3.51032 | 21.2474 | 54 | 36.09666 | 10.1939 | 61 | 13.19762 |
| Creatinine ROC | 0.29 | 7 | 0.488 | 0.08 | 61 | 0.277 | 0.18 | 65 | 0.391 |
| B2/creatinine | 0.3791 | 6 | 0.33655 | 1.1955 | 55 | 2.22057 | 0.6143 | 61 | 0.90479 |
| Vitamin B2 ROC | 0.3333 | 6 | 0.51640 | 0.4364 | 55 | 0.50050 | 0.3115 | 61 | 0.46694 |
| B2/creatinine ROC | 0.3333 | 6 | 0.51640 | 0.4364 | 55 | 0.50050 | 0.6230 | 61 | 0.48867 |
| Peak 2 area / Peak 1 area | 3.3471 | 6 | 1.59285 | 5.2637 | 54 | 7.48945 | 4.1196 | 61 | 4.86627 |
| Peak 2 amplitude / Peak 1 amplitude | 12.6634 | 6 | 12.61957 | 17.0468 | 54 | 24.52610 | 14.4725 | 61 | 29.08680 |
| Peak 2 amplitude - Peak 1 amplitude | 4.2625 | 6 | 3.03072 | 13.0804 | 54 | 20.57116 | 7.6109 | 61 | 10.32019 |
| Peak 2 area - Peak 1 area | 12.6304 | 6 | 8.27341 | 40.3259 | 54 | 66.85722 | 21.8185 | 61 | 29.97784 |
| (Peak 2 amplitude - Peak 1 amplitude) / creatinine | 0.8096 | 6 | 0.72408 | 2.5139 | 54 | 5.11354 | 1.5433 | 61 | 3.67045 |
| Peak 1 amplitude / Peak 2 amplitude | 0.3294 | 6 | 0.55104 | 0.2914 | 54 | 0.58291 | 0.3724 | 61 | 0.53113 |
| Peak 1 area / Peak 2 area | 0.4381 | 6 | 0.39856 | 0.4438 | 54 | 0.38774 | 0.5567 | 61 | 0.47255 |
| Peak 1 amplitude - Peak 2 amplitude | -4.2625 | 6 | 3.03072 | -13.0804 | 54 | 20.57116 | -7.6109 | 61 | 10.32019 |
| Peak 1 area - Peak 2 area | -12.6304 | 6 | 8.27341 | -40.3259 | 54 | 66.85722 | -21.8185 | 61 | 29.97784 |
| (Peak 1 amplitude - Peak 2 amplitude) / creatinine | -0.8096 | 6 | 0.72408 | -2.5139 | 54 | 5.11354 | -1.5433 | 61 | 3.67045 |
| (Peak 1 area - Peak 2 area) / creatinine | -3.3411 | 6 | 4.82111 | -7.5742 | 54 | 15.35162 | -4.3593 | 61 | 10.18464 |
| (Peak 1 area + Peak 2 area) / creatinine | 6.3855 | 6 | 8.44647 | 11.5180 | 54 | 19.37069 | 7.0372 | 61 | 11.92654 |
| Peak1 amplitude + Peak2 amplitude | 5.3979 | 6 | 2.99887 | 15.0544 | 54 | 21.24037 | 9.3372 | 61 | 10.64612 |
| (Peak 2 area - Peak 1 area) / creatinine | 3.3411 | 6 | 4.82111 | 7.5742 | 54 | 15.35162 | 4.3593 | 61 | 10.18464 |

**S 6. Significant Spearman's correlate relationships within the MTHFR 677 TT genotype.**

Admission frequency = admit no/DOI
Cost care burden = admission frequency + DSP
Correlation is generally significant at the 0.05 level (2-tailed).
Correlation is significant at the 0.01 level (2-tailed) (Bold text).
Red = over methylation markers
Blue – undermethylation markers
*The Benjamini-Hochberg procedure critical p values for Spearman correlations were 0.011 for the TT genotype, therefore all correlations in this table are significant after correction for multiple variables. DOI = duration of illness.

| **MTHFR 677 TT** | A<br>Dependent | A:B | B<br>Independent Correlate A with B | B:C | C<br>Correlate B with C Remarks |
|---|---|---|---|---|---|
| N 7, Prevalence 5.2%<br>Mean DOI 5.71 years | | | | | |
| | case | | **MTHFR 677 TT** | | |
| | case | N6, rho 0.828, P0.042 | Vitamin B2 ug/l | N 6, rho 0.829, P 0.042 | Peak 2 amplitude / Peak 1 amplitude |
| | case | N6, rho 0.828, P0.042 | **Vitamin B2/creatinine** | N6, rho 0.886, P 0.019 | Peak 2 area / Peak 1 area |
| | case | N6, rho 0.828, P0.042 | Vitamin B2/creatinine | | Peak 2 amplitude / Peak 1 amplitude |
| | case | N 6, rho 1.000, P 0.000 | **Vitamin B2/creatinine ROC** | | |
| | case | N6, rho 0.828, P 0.042 | **Peak2/Peak 1 amplitude** | | |
| | case | N6, rho 0.828, P 0.042 | **Peak2/Peak 1 area** | | |
| | case | **N 7, rho 1.000, P 0.000** | **High DA ROC** | | |
| | case | N 7, rho 0.750, P 0.052 | AD/NA ROC | | |

## S 7. Significant Spearman's correlate relationships within the MTHFR 677 CC genotype.

(P2/P1, P2-P1 = riboflavin synthesis and P1-P2 amplitude or area under peak = riboflavin (vitamin B2) metabolism products). In the interest of space, not statistically significant in the correlation matrix, have not been included and their absence as significant variables must be imputed from their absence.
Admission frequency = admit no/DOI
Cost care burden = admission frequency + DSP*. Correlation is significant at the 0.05 level (2-tailed). **. Correlation is significant at the 0.01 level (2-tailed) (Bold text).
Red = over methylation markers. Blue – undermethylation markers
(Benjamini-Hochberg critical p 0.039 for the CC genotype, DOI = duration of illness))

| **MTHFR 677 CC** | **Dependent A** | **A:B** | **Independent B** | **B:C** | **C** |
|---|---|---|---|---|---|
| **N 57-65,** Prev 48.5% | **Mean DOI 7.18** | | **Correlate A with B** | | **Correlate B with C** |
| | case | | **MTHFR 677 CC** | | |
| | case | N64, rho -0.245, P 0.051 | RC folate | | |
| | | | RC folate | N 62, rho 0.287, P 0.024 | Vitamin B6 |
| | **case** | **N 64, rho 0.323, P 0.009** | **Low RC folate ROC** | | |
| | case | **Nil sig** | **Vitamin B12/RC folate** | | |
| | **case** | **N63, rho 0.432, P 0.000** | **Low Vit B6 ROC** | | |
| | **case** | **N63, rho -0.335, P 0.007** | **Vitamin B6** | | |
| | | | Vitamin B6 | N 62, rho 0.287, P 0.024 | RC folate |
| | | | Vitamin B6 | N 61, rho -0.277, P 0.078 | NA/MHMA |
| | **case** | **N 64, rho -0.344, P 0.005** | **Low Vitamin D ROC** | | |
| | | | **Vitamin D** | N 63, rho 0.295, P 0.019 | RC FOLATE |
| | | | **Vitamin D** | Nil sig | Vitamin B6 |
| | | | **Vitamin D** | N 62, rho -0.322, P 0.011 | NA/MHMA |
| | **case** | **N 60, rho 0.475, P 0.000** | **5HIAA** | | |
| | **case** | **N65, rho 0.263, P 0.000** | **5HIAA ROC** | | |

| | | |
|---|---|---|
| case | N65, rho 0.263, P 0.034 | DA |
| **case** | **N65, rho 0.362, P 0.003** | **High DA ROC** |
| **case** | **N65, rho 0.634, P 0.000** | **NA** |
| **case** | **N65, rho 0.643, P 0.000** | **High NA ROC** |
| **case** | **N63, rho 0.552, P 0.000** | **NA/MHMA** |
| **case** | **N63, rho 0.556, P 0.000** | **High NA/MHMA ROC** |
| **case** | **N 65, rho 0.669, P 0.000** | **AD** |
| **case** | **N65, rho 0.616, P 0.000** | **High AD ROC** |
| **case** | **N65, rho 0.634, P 0.000** | **AD/MHMA** |
| case | **N65, rho 0.601, P 0.000** | **NA + AD/MHMA** |
| **case** | **N65, rho 0.650, P 0.000** | **High AD/MHMA ROC** |
| **case** | **N65, rho 0.361, P 0.003** | **AD/NA** |
| **case** | **N65, rho 0.430, P 0.000** | **AD/NA ROC** |

**S8. Cross tab analysis for MTHFR 677 TT dependent variables.**

.

```
tab case B2ROC if mthfrpoly== homozygous = MTHFR 677 TT
           |          B2ROC
      case |      Pos.        Neg. |     Total
-----------+----------------------+----------
  Abnormal |         2           0 |         2
    Normal |         0           4 |         4
-----------+----------------------+----------
     Total |         2           4 |         6

True abnormal diagnosis defined as case = 1
```

**S9. Logistic regression analysis for MTHFR 677 CC dependent biochemical variables** (taking into account population [prevalence of the disease (0.45%)).

```
Logistic regression
   MTHFR 677 CC    Obs 57, LR Chi2(4)45.72
Prob > chi2 0.0000, Pseudo R2 0.5797
. diagt case predcase10 if mthfrpoly==2
           |     RECODE of p10
           |(Pr(case))
      case |         Pos.         Neg. |       Total
    Normal |           2           25 |          27
  Abnormal |          28            2 |          30
    Total |          30           27 |          57
True abnormal diagnosis defined as case = 1

        case | Odds Ratio   Std. Err.      z     P>|z|      [95% Conf. Interval]
  Vitamin B6 |   .974799   .0122398    -2.03   0.042     .9511021     .9990863
Peak / Ratio |  .1738954   .1643242     1.85   0.064     .0272858     1.108255
 AD + NA/MHMA|   1.25044   .0909248     3.07   0.002     1.084347     1.441974
      5-HIAA |  1.816841   .6930404     1.57   0.118     .8602464     3.837167
       _cons |  .2041357   .3062405    -1.06   0.290     .0107885     3.862558

Loglikelihood-16.572295              {95% Confidence Interval]
--------------------------------------------------------------------
Prevalence                      Pr(A)       .45%    ----- (given) -----
--------------------------------------------------------------------
Sensitivity                    Pr(+|A)     93.3%      77.9%     99.2%
Specificity                    Pr(-|N)     92.6%      75.7%     99.1%
ROC area              (Sens. + Spec.)/2      .93       .862      .997
--------------------------------------------------------------------
Likelihood ratio (+)     Pr(+|A)/Pr(+|N)    12.6       3.31        48
Likelihood ratio (-)     Pr(-|A)/Pr(-|N)    .072      .0188      .276
Odds ratio                   LR(+)/LR(-)     175       24.8      1234
Positive predictive value        Pr(A|+)   5.39%      1.47%     17.8% (lr)
Negative predictive value        Pr(N|-)    100%      99.9%      100% (lr)
--------------------------------------------------------------------
Pre-test odds                prev/(1-prev)   .0045    ----- (given) -----
Post-test odds (+)    Pr(A|+)/(1-Pr(A|+))    .057      .015      .217  (lr)
Post-test odds (-)    Pr(A|-)/(1-Pr(A|-))  .00033    .00125   8.5e-05 (lr)
```

*z is coefficient/Standard error (Std. Err.), where OR (Odds Ratio) is exponential of the coefficient of predictor variables.
Pz = probability associated z factor at 95% confidence level. Peak ratio = Peak 1 amplitude / Peak 2 amplitude.

**<u>Logistic regression for biomedical variables, without HPLC data.</u>**

**MTHFR 677 CC**

| | | |
|---|---|---|
| Number of obs | = | 59 |
| LR chi2(4) | = | 43.9 |
| Prob > chi2 | = | 0 |
| Pseudo R2 | = | 0.5369 |

Log likelihood = -18.934775

| case certainty CC n 59 | Odds Ratio (OR) | Std. Err. | z | P>\|z\| | [95% Conf. | Interval] |
|---|---|---|---|---|---|---|
| **free % Cu/Zn ROC** | 9.975525 | 11.82251 | 1.94 | 0.052 | 0.9775362 | 101.7979 |
| **AD / MHMA ROC** | 25.40205 | 24.09701 | 3.41 | 0.001 | 3.957291 | 163.057 |
| **Vitamin B12 /vitanin D ROC** | 21.29819 | 25.73367 | 2.53 | 0.011 | 1.994681 | 227.4113 |
| **Low vitamin B6 ROC** | 12.17347 | 12.51401 | 2.43 | 0.015 | 1.623309 | 91.2909 |
| _cons | 0.0020782 | 0.0037968 | -3.38 | 0.001 | 0.0000579 | 0.0746153 |

**S 10. Significant case- correlates within the MTHFR 677 CT genotype**

Correlation is significant at the 0.05 level (2-tailed). **. Correlation is significant at the 0.01 level (2-tailed) (Bold text). Red = overmethylation markers. Blue – undermethylation markers

(Critical Benjamini-Hochberg correction P value is 0.038 for the CT genotype).

| MTHFR 677 CT | Dependent A | A:B | Independent B | B:C | Interdependent C |
|---|---|---|---|---|---|
| | case | 40/42 significant symptoms | Symptoms | | |
| | case | N 61, rho 0.384, P 0.002 | DA | | |
| | case | N 61, rho 0.377, P 0.003 | High DA ROC | | |
| | case | N61, rho0.686, P 0.000 | NA | | |
| | case | N 61, rho 0.642, P 0.000 | High NA ROC | | |
| | case | N 61, rho 0.591, P 0.000 | AD | | |
| | case | N 61, rho 0.607, P 0.000 | High AD ROC | | |
| | case | N 60, rho 0.545, P0.000 | NA/MHMA | | |
| | case | N 60, rho 0.567, P 0.000 | NA/MHMA ROC | | |
| | case | N 60, rho 0.427, P 0.001 | AD/MHMA | | |
| | case | N60, rho 0.528, P 0.000 | AD/MHMA ROC | | |
| | case | N 61, rho 0.498, P0.000 | NA/DA | | |
| | case | N 61, rho 0.481, P 0.000 | NA/DA ROC | | |
| | case | N 61, rho 0.339, P 0.007 | AD/NA ROC | | |
| | case | N 54, rho 0.382, P 0.004 | Area under peak 2 ROC | | |
| | case | N 54, rho 0.316, P 0.020 | Peak 1-peak 2 area ROC | | |

| | | | | |
|---|---|---|---|---|
| case | Nil sig | % free Cu/Zn | | |
| case | N 62, rho 0.323, P 0.011 | Low folate ROC | | |
| | | Vitamin B12 | Nils sig | RC folate |
| | | Vitamin B12 | N 54, rho 0.339, P 0.012 | Peak 2 amp/peak 1 amp |
| | | Vitamin B12 | N 54, rho 0.329, P 0.015 | Peak 2 area/peak1 area |
| case | N 62, rho 0.228, P 0.075 | High vit B12 ROC | | |
| case | N 62, rho 0.277, P 0.029 | [Vitamin B12 X % free Cu X /[Zinc X folate] | | |
| case | N 58, rho 0.294, P 0.025 | [Vitamin B12 X % free Cu X homocysteine]/ | | |
| case | N 57, rho 0.277, P 0.037 | [Vitamin B12 X %free Cu X homocysteine] / [Zn X folate X vitamin B6 X vitamin D] | | |
| case | Nil sig (cancels) | Vitamin B6? | | |
| | | Vitamin B6 | N 59, rho 0.373, P 0.004 | RC folate |
| | | Vitamin B6 | N 53, rho 0.449, P 0.001 | B2 ug/L |
| | | Vitamin B6 | N 53, rho 0.314, P 0.022 | B2/creatinine |
| | | Vitamin B6 | N 52, rho 0.306, P 0.027 | Peak 2 amp/peak 1 amp |
| | | Vitamin B6 | N 52, rho 0. 316, P 0.023 | Peak 2 area/peak1 area |
| | | Vitamin D | N 61, rho 0.252, P 0.050 | RC folate |
